# Dyadic mobile intervention empowering lifestyle modification in chronic kidney disease management: A feasibility randomized controlled trial

**DOI:** 10.1016/j.ijnsa.2026.100549

**Published:** 2026-05-14

**Authors:** Chun-Yi Ho, Deborah Siregar, Wei-Hung Lin, Junne-Ming Sung, Ming-Cheng Wang, Miaofen Yen

**Affiliations:** a Department of Nursing, College of Medicine, National Cheng Kung University, Tainan City, Taiwan; b Department of Internal Medicine, National Cheng Kung University Hospital, College of Medicine, National Cheng Kung University, Tainan City, Taiwan

**Keywords:** Caregivers, Feasibility studies, Healthy lifestyle, Mobile health, Renal Insufficiency, Chronic

## Abstract

**Background:**

Sustaining lifestyle modification is essential for managing chronic kidney disease. Dyadic mobile interventions engaging patients and their significant others (a spouse or adult child) may offer potential support, yet their feasibility in clinical nursing practice requires evaluation.

**Objective:**

To evaluate the feasibility, acceptability, and safety of a clinic-integrated Digital Dyadic Empowerment Program to inform a full-scale trial.

**Design:**

A two-arm, parallel-group randomized controlled trial.

**Settings:**

Nephrology outpatient clinics at a medical center in Taiwan.

**Participants:**

Sixty patient-caregiver dyads (120 individuals).

**Methods:**

Dyads were randomized 1:1 to receive a staff-assisted mobile intervention providing lifestyle support plus usual care (intervention group), or usual care alone (control group) for 3 months. Feasibility criteria included: recruitment of ≥ 30 dyads monthly, study completion rate ≥ 80%, average 90-day platform usage rate (use days/90) ≥ 50%, and adverse event rate < 5%.

**Results:**

The recruitment target was met in 46 days (60 dyads from 2214 screened), the efficiency was ∼39 dyads per month. The retention rate was high at 91.7% (55[26+29]/60, 95% CI [81.9, 96.4]), with attrition primarily linked to older age and caregiver burden. Platform uptake was 100%, but the 90-day platform usage rates averaged 48.93% (1145/2340[26 × 90], 95% CI [46.91, 50.96]) among completers. A bimodal pattern emerged: 14 high-usage dyads averaged 87.06% (1097/1260[14 × 90], 95% CI [85.10, 88.80]) while 12 low-usage dyads averaged 4.44% (48/1080[12 × 90], 95% CI [3.37, 5.84]). No adverse events occurred (0%, 0/60, 95% CI [0.0, 6.0]), but 1 intervention dyad withdrew due to psychological burden of daily self-monitoring.

**Conclusions:**

Digital Dyadic Empowerment Program is safe and feasible to integrate into routine care, demonstrating efficient recruitment and high retention. However, variable engagement and potential burden highlight the need for tailored nursing support and refined onboarding procedures. A full-scale trial will proceed using our proposed protocol adaptations.

**Registration:**

ClinicalTrials.gov, NCT06226649. Registered January 18, 2024; first recruitment January 29, 2024.

**What is already known**

• Sustaining lifestyle modifications is a cornerstone of chronic kidney disease management but remains a significant challenge for patients and their families.
• Digital health interventions show promise for supporting self-management, but their effectiveness can be limited by low engagement and poor integration into clinical workflows.
• Involving caregivers or family members (a Dyadic approach) may improve patient adherence and outcomes in chronic illness, but this model is not yet commonly applied or rigorously tested.

**What this paper adds**

• Demonstrates the feasibility of recruiting and retaining patient-caregiver dyads from a busy clinical setting for a digital health trial, achieving a high completion rate of 91.7%.
• Provides objective evidence on the engagement patterns with a Dyadic messaging app, revealing a bimodal usage pattern of “high-users” and “low-users” that informs strategies for future adaptive interventions.
• Proposes a detailed protocol for adaptations needed for a future definitive randomized controlled trial and for related research, including strategies to improve user engagement and reduce participant burden.

Alt-text: Unlabelled box dummy alt text

## 1 Introduction

### 1.1 Background

Chronic kidney disease represents a significant and growing global health burden, affecting approximately 700 million people worldwide (2017 estimates) (GBD Chronic Kidney Disease Collaboration, 2020). This condition contributes to premature mortality, reduced quality of life, and substantial costs for health systems and families (Francis et al., 2024). Clinical guidelines highlight lifestyle factors and dietary patterns as core components of chronic kidney disease care, and recommend regular physical activity for most people with non-dialysis chronic kidney disease (Baker et al., 2022; Kidney Disease: Improving Global Outcomes (KDIGO) CKD Work Group 2024). However, effective lifestyle modification usually requires continuous self-regulation outside clinical settings. People living with chronic kidney disease, particularly older adults, often face psychosocial and contextual barriers, alongside health-system constraints in care delivery (Cardol et al., 2022; Hirsch et al., 2024).

Digital health interventions offer a scalable solution to bridge this gap by supporting continuous self-management (Daniels and Bonnechère, 2024; Jeddi et al., 2017). Yet, many digital tools face barriers to user engagement. Patients experiencing digital poverty and other structural disadvantages are less likely to access and benefit from these interventions, and implementation into routine care pathways remains challenging (Graham-Brown et al., 2022; Mumtaz et al., 2023). Accordingly, contemporary chronic care models increasingly emphasize family involvement and shared self-management, aligning with nursing priorities in supporting patients within their everyday social context (Martire and Helgeson, 2017). Informal caregivers, typically family members, are frequently involved in day-to-day illness management and may influence patients’ psychosocial adjustment during long-term self-care (Shaffer et al., 2020). Digital interventions adopting a Dyadic approach, which actively engages both the patient and a significant other (e.g., a spouse, adult child, or close friend), may be a promising strategy to improve adherence to lifestyle modification but requires further investigation.

### 1.2 Previous work and rationale

Our prior randomized trial demonstrated that engaging significant others through helping-relationship strategies was associated with improvements in health-promoting lifestyles among people with chronic kidney disease (Chao et al., 2022a). In Taiwan, LINE dominates the instant messaging market, accounting for over 90% market share among internet users (Taiwan Network Information Center, 2024), making it a practical channel for delivering dyadic digital health interventions. The Digital Dyadic Empowerment Framework was therefore proposed to guide the design of digital interventions that support sustainable lifestyle modification among chronic kidney disease dyads. Following this framework, a LINE-based digital health intervention platform with an extended application, “*Kidney Lifestyle*,” was developed and completed initial usability testing (Ho et al., 2025).

Although the Digital Dyadic Empowerment Program has been established technically, the feasibility of integrating this Dyadic intervention into busy outpatient nursing workflows remains unexamined. A feasibility study is therefore warranted to evaluate key uncertainties (e.g., recruitment, retention, engagement, and safety) and to ensure implementation protocols are robust before a full-scale randomized controlled trial (Eldridge et al., 2016).

### 1.3 Aims

The primary aim of this study was to evaluate the feasibility of the Digital Dyadic Empowerment Program—a clinic-integrated mobile intervention designed to support lifestyle modification in patient-caregiver dyads. The findings will determine the viability of the protocol and inform the design of a future definitive randomized controlled trial. A secondary aim was to explore preliminary effects on clinical and psychosocial outcomes to inform the sample size calculation and analysis plan for a future trial.

## 2 Methods

### 2.1 Study design

This study was a two-arm, parallel-group, randomized controlled trial with a 1:1 allocation ratio. The reporting adheres to the Consolidated Standards of Reporting Trials (CONSORT) extension for pilot and feasibility trials (Eldridge et al., 2016) and the CONSORT-EHEALTH guidelines (Eysenbach and CONSORT-EHEALTH Group, 2011). The study protocol was prospectively registered on January 18, 2024 at ClinicalTrials.gov (Identifier: NCT06226649).

### 2.2 Participants and setting

Participants were recruited between January and March 2024 from outpatient nephrology clinics at a medical center in Tainan, Taiwan. In this clinical setting, patients typically attend follow-up appointments every 1 to 3 months for usual care (detailed in 2.3.2.), either independently or accompanied by significant others. The recruitment was carried out by three nephrologists and two research assistants. Eligible criteria for dyads, consisted of a patient and a significant other, were as follows:

• Inclusion criteria: (1) patient aged ≥ 20 years with a diagnosis of chronic kidney disease for ≥ 6 months; (2) patient able to identify a significant other willing to participate; and (3) both members able to communicate in Mandarin or Taiwanese.
• Exclusion criteria: (1) significant other being a healthcare provider; (2) either member having a diagnosed mental illness; (3) patient receiving renal replacement therapy; or (4) either member lacking access to a smartphone or not using the LINE instant messaging app.

During outpatient visits, nephrologists screened patients attending with a significant other and referred potential dyads to a research assistant for eligibility confirmation. The research assistant then invited eligible dyads and provided study information, including required baseline and follow-up assessments. Intervention-group dyads additionally received instructions for joining and using the Digital Dyadic Empowerment Program platform.

### 2.3 Interventions

#### 2.3.1 Digital Dyadic empowerment program (Intervention group only)

Dyads in the intervention group received lifestyle modification instant-messaging support through a LINE Official Account named “*Kidney Lifestyle*” and its extended application. The digital platform is accessible to all LINE users by adding the Official Account (ID: @509kgajt). Educational content provided by the platform was previously reviewed and approved by health educators from the Health Education Center at National Cheng Kung University Hospital, nephrologists, and healthcare experts. Use was free of charge and educational materials are publicly viewable within the platform.

The primary objective of the intervention was to encourage dyads in modifying health-related lifestyles. Specifically, users were encouraged to engage daily with the following two main features to document their progress:

• Record Values: Allowing users to track physiological measurements (e.g., blood pressure) at home through the extended application.
• Kidney Diary: Facilitating daily recording of diet, medication intake, and physical activity through the extended application.

**Fig. 1:**
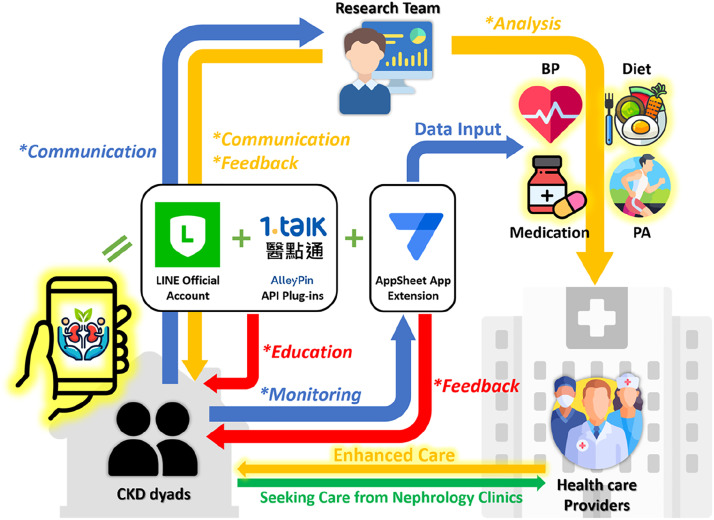
The schematic diagram of the intervention digital platform. CKD: chronic kidney disease; BP: blood pressure; PA: physical activity. The arrows marked with an asterisk (*) represent the practical application of the five digital strategies mentioned in the Digital Dyadic Empowerment Framework. Blue arrows represent the active engagement from dyads. Orange arrows denote service flows involving human support, while red arrows denote service flows with full automation. Reproduced from Ho et al. (2025), under the Creative Commons Attribution (CC BY 4.0) license.

Research assistants provided human support, including an initial onboarding session to set up the platform and subsequent online/offline support, with scheduling of motivational messages celebrating daily completion or push-notification messages triggered by interruptions lasting >2 days to encourage engagement. The intervention pathway is illustrated in Fig. 1.

#### 2.3.2 Usual care (Intervention and control groups)

All study participants received regular checkups and treatments at the nephrology clinics of National Cheng Kung University Hospital. Nephrologists diagnosed and explained disease progression, treatment plans, prognosis, and risks based on clinical assessments. Care plans included medication adherence, home blood pressure monitoring, dietary adjustments, and regular physical activity. Participants also received nephrology nursing or nutritional counseling through the hospital’s Health Education Center.

### 2.4 Outcomes

#### 2.4.1 Primary feasibility outcomes

Four feasibility outcomes were assessed against predefined criteria:

1. Recruitment Efficiency: Target of ≥ 30 dyads recruited per month.
2. Study Completion Rate: Target of ≥ 80% of participants completing the 3-month study in both groups.
3. Intervention Acceptability: Target of ≥ 50% average platform usage (daily use of either ‘Record Values’ or ‘Kidney Diary’ features) over 90 days.
4. Adverse Event Rate: Target of < 5% of participants reporting serious adverse events.

Research assistants monitored feasibility indicators throughout recruitment and follow-up, and summarized them after data collection completion.

#### 2.4.2 Secondary clinical outcomes

Secondary outcomes were collected at baseline and 3 months to explore preliminary effects. These included kidney function (estimated Glomerular Filtration Rate and its corresponding stage of chronic kidney disease) and patient-reported outcomes measures, including the stage of change based on the Transtheoretical Model, the Health-Promoting Lifestyle Profile-II (Teng et al., 2010), the Adherence to Healthy Behaviors Scale (Huang et al., 2021), the Dyadic Adjustment Scale-7 (Hunsley et al., 2001), the Helping Relationships from Significant Others scale (Chao et al., 2022b), and the World Health Organization Quality of Life-BREF (Yao et al., 2002). Definitions, scoring procedures, and data-collection details for each secondary clinical outcome are provided in the Supplementary Appendix.

### 2.5 Sample size

As a feasibility trial, no formal power calculation was performed to detect treatment effects. A sample size of 60 dyads (30 per group) was chosen as a practicable target to obtain stable estimates for the primary feasibility metrics (e.g., recruitment and completion rates) and to estimate the variability of clinical outcomes, consistent with established guidance for feasibility trials (Eldridge et al., 2016; Hertzog, 2008).

### 2.6 Randomization and blinding

A 1:1 simple randomization sequence was computer-generated using R software prior to recruitment by a research assistant. The sequence was uploaded to an administrative AppSheet application under the supervision of the principal investigator. Another research assistant not involved in sequence generation conducted participant enrollment by accessing the group allocation for each dyad after assigning them a sequential identification number.

Healthcare providers delivering usual care were blinded to group allocation. Research assistants, who provided onboarding and support, and participants could not be blinded due to the nature of the intervention. However, to minimize expectation bias and compensatory rivalry, participants were unaware of the alternative arm’s existence.

### 2.7 Statistical analysis

Primary feasibility outcomes were analyzed using descriptive statistics, including numbers, percentages, and 95% confidence intervals where appropriate. For the exploratory analysis of clinical outcomes, an intention-to-treat principle was followed (Ahn and Kang, 2023). Pre-post changes between groups were estimated using Generalized Linear Mixed Models (IBM, 2021; Sharpe and Cribbie, 2023). Due to the feasibility nature of the study, these analyses were considered descriptive, and statistical significance was not the primary focus of interpretation. All statistical analyses were performed using IBM SPSS Statistics, version 25.0. Full specifications for the exploratory clinical outcome analyses (model structure, handling of missing data, and per-protocol/sensitivity analyses) are provided in the Supplementary Appendix.

### 2.8 Ethical considerations

This study was approved by the Institutional Review Board of National Cheng Kung University Hospital, Tainan, Taiwan (IRB No B-ER-110-110). Before participation, all participants were fully informed about the study’s purpose, procedures, data protection measures, and their rights. Participation was voluntary, and participants could withdraw at any time without consequence. Written informed consent was obtained from both members of each dyad, who each received a signed copy of the consent form and a gift voucher of NTD $200 (approximately USD $6) for each completed assessment. All methods were carried out in accordance with the ethical principles of the Declaration of Helsinki, Taiwan’s Human Subjects Research Act, and applicable local regulations on human-subject research.

## 3 Results

### 3.1 Participant flow and baseline characteristics

**Fig. 2:**
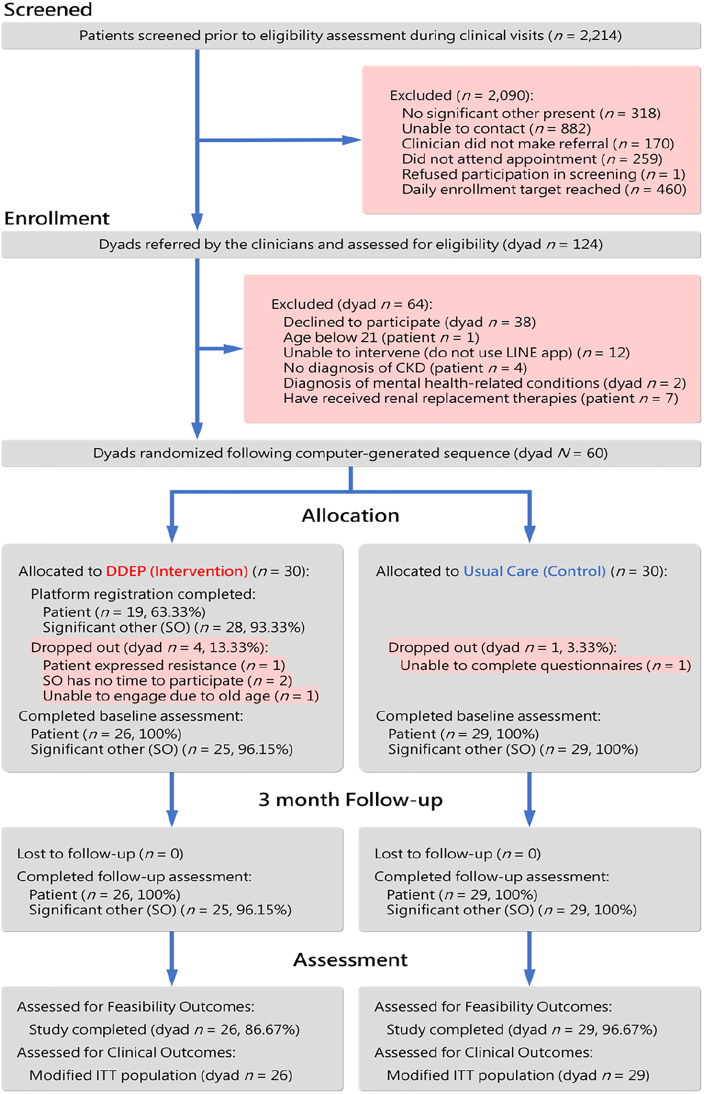
Consolidated standards of reporting trials (CONSORT) flow diagram. CKD: chronic kidney disease; ITT: intention-to-treat.

The flow of participants through the trial, from screening to final analysis, is detailed in the CONSORT diagram (Fig. 2). Of 2214 patients screened during clinic visits, 124 dyads were referred for eligibility assessment, and 60 were randomized. A total of 55 dyads (91.7%) completed the 3-month study. The baseline demographic and clinical characteristics of the 55 patients who completed the study were broadly similar between the two groups (Table 1).

**Table 1:** Baseline demographic and clinical characteristics of patient participants. a, b.

Variable	All (*N* = 55)	Intervention (DDEP + UC) (*n* = 26)	Control (UC only) (*n* = 29)
**Age**^a^	68.73 (12.49)	68.69 (10.45)	68.76 (14.26)
**Sex**^b^			
Male	33 (60.0)	13 (50.0)	20 (69.0)
Female	22 (40.0)	13 (50.0)	9 (31.0)
**Education**^b^			
Junior high or below	23 (41.8)	12 (46.2)	11 (37.9)
High school/vocational	14 (25.5)	3 (11.5)	11 (37.9)
College or above	18 (32.7)	11 (42.3)	7 (24.1)
**Marital Status**^b^			
Married	48 (87.3)	23 (88.5)	25 (86.2)
Unmarried	7 (12.7)	3 (11.5)	4 (13.8)
**Employment**^b^			
Yes	15 (27.3)	5 (19.2)	10 (34.5)
No/retired	40 (72.7)	21 (80.8)	19 (65.5)
**Role of SO**^b^			
Spouse	26 (47.3)	10 (38.5)	16 (55.2)
Child/grandchild	27 (49.1)	15 (57.7)	12 (41.4)
Other relative	2 (3.6)	1 (3.8)	1 (3.4)
**eGFR**^a^	45.84 (26.03)	46.29 (21.65)	45.43 (29.79)
**CKD Stage**^b^			
Stage 1	3 (5.5)	2 (7.7)	1 (3.4)
Stage 2	11 (20.0)	4 (15.4)	7 (24.1)
Stage 3a	14 (25.5)	8 (30.8)	6 (20.7)
Stage 3b	9 (16.4)	5 (19.2)	4 (13.8)
Stage 4	11 (20.0)	5 (19.2)	6 (20.7)
Stage 5	7 (12.7)	2 (7.7)	5 (17.2)
**Stage of Change**^b^			
Precontemplation	3 (5.5)	2 (7.7)	1 (3.4)
Contemplation	1 (1.8)	0 (0.0)	1 (3.4)
Preparation	6 (10.9)	5 (19.2)	1 (3.4)
Action	14 (25.5)	7 (26.9)	7 (24.1)
Maintenance	31 (56.4)	12 (46.2)	19 (65.5)
**HPLP-II**^a^	141.40 (23.04)	142.19 (24.8)	140.69 (21.7)
Health responsibility	25.16 (5.23)	24.23 (5.4)	26.00 (5.1)
Physical activity	19.69 (5.43)	19.08 (5.6)	20.24 (5.3)
Nutrition	24.64 (4.31)	25.08 (4.7)	24.24 (4.0)
Spiritual growth	25.58 (5.74)	26.12 (5.8)	25.10 (5.7)
Interpersonal relations	24.44 (4.92)	25.54 (4.7)	23.45 (5.0)
Stress management	21.89 (4.28)	22.15 (3.8)	21.66 (4.7)
**AHBS**^a^	45.09 (5.68)	45.23 (6.0)	44.97 (5.5)
Abstinence	14.07 (3.64)	14.77 (2.4)	13.45 (4.4)
Recommendations	20.76 (2.93)	20.73 (2.8)	20.79 (3.1)
Precautions	10.25 (2.30)	9.73 (2.8)	10.72 (1.7)
**WHOQOL-BREF**^a^			
Physical	13.87 (2.62)	13.82 (3.0)	13.91 (2.3)
Psychological	13.77 (2.41)	14.08 (2.7)	13.49 (2.2)
Social	13.98 (2.22)	14.19 (2.2)	13.79 (2.2)
Environment	14.57 (2.10)	14.63 (2.2)	14.51 (2.0)
**DAS-7**^a^	21.36 (6.46)	20.58 (6.8)	22.07 (6.1)
**HRSO**^a^	89.56 (15.14)	88.58 (18.2)	90.45 (12.0)
Understanding	34.95 (6.99)	34.19 (8.6)	35.62 (5.2)
Caring	37.27 (5.90)	36.81 (7.2)	37.69 (4.5)
Coaching	17.35 (3.81)	17.58 (3.8)	17.14 (3.9)

*Notes:* DDEP: Digital Dyadic Empowerment Program; UC: usual care; SO: significant other; eGFR: estimated Glomerular Filtration Rate; CKD = chronic kidney disease; HPLP-II: Health-Promoting Lifestyle Profile-II; AHBS: Adherence to Healthy Behaviors Scale; WHOQOL-BREF: World Health Organization Quality of Life-BREF questionnaire; DAS-7: Dyadic Adjustment Scale-7; HRSO: Helping Relationships from Significant Others scale.

a Mean (standard deviation)

b Number (%).

### 3.2 Feasibility outcomes

#### 3.2.1 Recruitment efficiency

A total of 60 dyads were successfully recruited over a 46-day period (January 29 to March 14, 2024), yielding a recruitment rate of approximately 39 dyads per month. This exceeded the predefined criterion of ≥ 30 dyads per month. The referral rate was 5.6%, as 124 dyads were referred from 2214 patients screened. The primary reasons for exclusion during physician screening were research staff being unable to contact patients due to other commitments (42.2% [882/2090]), the daily enrollment target being reached (22.0% [460/2090]), or the patient not being accompanied by an significant other (15.2% [318/2090]). The enrollment (consent) rate was 48.4% (60/124), with main reasons for exclusion being the dyad declining to participate (59.4% [38/64]) or not using the LINE application (18.8% [12/64]).

#### 3.2.2 Study completion rate

Fifty-five of the 60 randomized dyads completed the 3-month follow-up, resulting in an overall study completion rate of 91.7% (55/60, 95% CI [81.9, 96.4]). The completion rate was 86.7% (26/30) in the intervention group and 96.7% (29/30) in the control group, both surpassing the predefined success criterion of ≥ 80%. Follow-up assessments were collected between April 22 and November 8, 2024. The 4 intervention dyads withdrew due to reasons including the significant other having no time (*n* = 2), the patient expressing resistance (*n* = 1), or unable to engage due to old age (*n* = 1). The 1 control dyad withdrew due to the perceived complexity of the questionnaires.

#### 3.2.3 Intervention acceptability

**Fig. 3:**
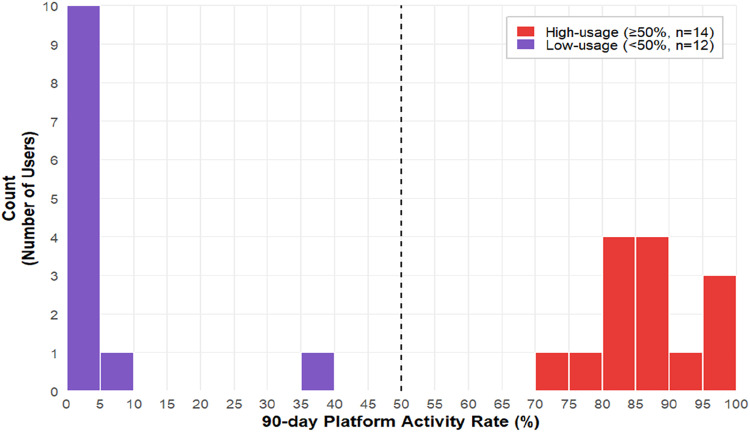
Distribution of 90-day platform activity rate among participants in the intervention group (modified intention-to-treat population, *n* = 26).

Among the 30 intervention dyads, 19 patients (63.3%,95% CI [45.51, 78.13]) and 28 significant others (93.3%, 95% CI [78.68, 98.15]) registered on the platform. Every dyad had ≥ 1 member registered, and 17 dyads (56.67%, 95% CI [39.20, 72.62]) had both members registered. For the 26 completers, the 90-day platform usage rate averaged 48.93% (1145/2340[26 × 90] days, 95% CI [46.91, 50.96]), just below the predefined criterion of 50%. Platform usage demonstrated a bimodal pattern with two subgroups: a high-usage group of 14 dyads with an average usage rate of 87.06% (1097/1260[14 × 90], 95% CI [85.10, 88.80]), and a low-usage group of 12 dyads with an average usage rate of 4.44% (48/1080[12 × 90], 95% CI [3.37, 5.84]) (Fig. 3).

#### 3.2.4 Safety (Adverse event rate)

No serious adverse events were reported during the trial, resulting in an adverse event rate of 0% (0/60, 95% CI [0.0, 6.0]). This met the predefined safety criterion of < 5%. One dyad withdrew from the intervention group due to self-reported psychological burden associated with patient’s resistance to daily monitoring, but this was not classified as a serious adverse event.

### 3.3 Exploratory analysis of clinical outcomes

The mean change in kidney function indicated a smaller decline in the intervention group compared to the control group. Across most continuous self-report measures, the intervention group showed small, non-significant improvements from baseline to follow-up, while the control group showed slight declines. However, the 95% confidence intervals for these within-group differences consistently included zero. Detailed results for each clinical outcome are available in the Supplementary Appendix (Tables S1-S24).

## 4 Discussion

### 4.1 Principal findings

The study met three of its four predefined feasibility criteria. Recruitment efficiency was high, with 60 dyads enrolled in 46 days (∼39 dyads/month). The study completion rate was 91.7%, well above the 80% target. The intervention was safe, with a 0% rate of serious adverse events. The fourth criterion, intervention acceptability, was narrowly missed, with a mean usage rate of 48.93% against a 50% target. However, the identification of a bimodal usage pattern (high vs. low engagement) provides critical information for refining the intervention and analysis plan for a full-scale trial.

### 4.2 Comparison with prior work

Across recent nephrology digital health feasibility trials, recruitment throughput and follow-up completion have been variable, with completion rates commonly below 90% (Dawson et al., 2021; Lightfoot et al., 2024; Reston et al., 2023; Young et al., 2024). In this feasibility trial, we enrolled ∼39 dyads per month and retained 91.7% of dyads to the follow-up assessment. Notably, this was achieved despite a low physician referral rate (5.6% [124/2214]), suggesting that staffing and workflow capacity may be important practical constraints for future scale-up. The observed high follow-up completion may be partly attributable to embedding study procedures within routine clinical care and the supportive nature of the dyadic design.

In response to prior evidence of reduced adherence in long-duration digital lifestyle interventions (Kelders et al., 2012), we selected structured daily diary-style interactions as a pragmatic strategy to encourage frequent engagement. Using our day-level definition of engagement (i.e., any platform activity recorded on a given day), the overall adherence was moderate (∼49%). The bimodal usage pattern (Fig. 3) mirrors Eysenbach’s (2005) observation of “*hardcore users*” versus “*nonusers*” in eHealth usage behavior. Importantly, the observed divergence may be shaped not only by individual preferences but also by potentially modifiable determinants within clinical implementation, such as the quality of onboarding, the stability of support from significant others, and the usability burden experienced during daily self-monitoring. This interpretation is consistent with prior chronic kidney disease digital interventions reporting pragmatic barriers to engagement (e.g., time constraints) and the need for implementation adaptations to sustain participation (Dawson et al., 2021; Young et al., 2024). Furthermore, future studies could explore the “time since diagnosis” as a potential factor influencing engagement, as patients in different stages of change may exhibit varying levels of adherence to digital monitoring. Consequently, a key objective for future definitive trials is to enhance engagement among potential nonusers. This may be achieved by adopting strategies consistent with the principles of Just-in-Time Adaptive Interventions (Nahum-Shani et al., 2018).

Finally, while some prior trials in kidney-related populations reported few or no intervention-related adverse events (Brown et al., 2024; Young et al., 2024), our trial highlights potential non-physical harms that may accompany intensive self-monitoring. We identified psychological burden as a tangible adverse effect, exemplified by a dyad withdrawing due to anxiety and perceived pressure related to daily monitoring. To reduce distress, nursing professionals are encouraged to position the platform as a supportive tool for self-monitoring and to address concerns that it may feel like surveillance.

### 4.3 Strengths and limitations

This study has several strengths: (1) recruitment was conducted in a real-world clinical setting to reach the targeted population; (2) a rigorous feasibility methodology with prespecified criteria was used; (3) the intervention was delivered via LINE, a widely used messaging platform in Taiwan, ensuring ecological validity and distinguishing our program from fully automated apps through individualized two-way communication; (4) the innovative dyadic approach leveraged family support systems; and (5) engagement was measured precisely to identify usage patterns.

Limitations include: (1) the single-center design and potential selection bias may limit representativeness; (2) the level of significant other involvement was variable and not separately analyzed; (3) high variability in engagement could dilute treatment effects in a future trial; and (4) usage patterns were not analyzed temporally to understand changes in engagement over time and to explore potential threats to internal validity, such as the novelty and Hawthorne effects (McCambridge et al., 2014; Shin et al., 2018).

## 5 Implications for nursing

A dyadic digital health intervention like “*Kidney Lifestyle*” may serve as a powerful tool to extend care beyond the clinic. With such a platform, nurses in nephrology settings can facilitate more proactive and personalized care to reinforce education, monitor self-management behaviors between visits, and provide continuous support for patients and their significant others.

The lessons learned from this feasibility study provide a clear roadmap for a definitive trial. Adaptations have been identified across all feasibility domains to optimize the study protocol, intervention delivery, and analytical strategy. Table 2 summarizes the results, implications, and proposed adaptations.

**Table 2:** Digital Dyadic empowerment feasibility trial results, implications or issues identified, and proposed adaptations for the future definitive trial.

Results	Implications or Issues Identified	Proposed Adaptations for the Definitive DDEP Trial
**Recruitment** • 60 dyads in 46 days (∼39 dyads per month) • Referral rate = 5.6% (124/2214) • Consent (enrollment) rate = 48.4% (60/124)	• Clinic-based recruitment ensured reach to intended population • Despite low referral rate, recruitment pace is promising for scale-up	• No major changes required • If recruitment lags, providing additional staff would ensure adequate availability
	• Refusal is common due to perceived participation burden • Better avoid complex research protocols	• Provide visual study brief to reduce perceived uncertainty • Increase motivation: emphasize potential benefits and share success stories • Allow sufficient time for Q&A to clarify doubts
**Study Completion** • Completion rate = 91.67% (55/60) • 5 dyads withdrew (4 Int., 1 Control)	• Dropouts largely related to older ages and caregiver burden • Expectation management must start at consent and continue throughout the study	• Provide one-page study summary with contact details • Keep phone/LINE consultation channels open
	• There is a trade-off between study completion and heavy data collection	• Reduce questionnaires: retain HPLP-IICR and DAS-7 (∼68% reduction) • Require on-site completion to avoid lost follow-up
**Intervention Acceptability** • Mean 90-day platform usage rate = 48.93% (1145/2340) among 26 Int. users • Bimodal usage pattern: High/low subgroups identified (∼87% vs. ∼4%)	• High users suggest feasibility of integrating DDEP with usual care to promote home-based lifestyle modification • Nearly half nonusers highlight the need to improve onboarding, SO support, and usability • Engagement measures must avoid backfiring—reminders should be adaptive and well-timed • Daily usage tracking (including calculation of continuous vs. interrupted days) can support just-in-time interventions	*Increase Engagement* • “First-step” encouragement message after first log • Automated diet feedback (flag CKD restricted food) • User report summarizing diet/medication/activity/BP for dyads & clinicians • More varied, novel reminders (stickers, phone calls)
		*Reduce Fatigue* • Cancel default automated reminders and allow opt-in • If ≥2 days without logging, trigger reminders with direct links to log page • If ≥7 days inactive, send weekly care-call invitations (adjust timing by receptivity)
**Adverse Event** • Serious AEs = 0% (0/60) • One dyad reported psychological burden from daily health monitoring request	• DDEP is safe: with no physical harm and with enhanced data security • Daily self-monitoring may cause pressure	• At enrollment, educate BOTH patient and SO that app is supportive, not surveillance • Reassure that non-use and opt-out anytime is acceptable when feeling pressured
**Clinical Outcomes** • Compared with Controls, Int. patients showed a smaller eGFR decline and greater self-reported outcome score improvements	• The 95% CIs of pre-post contrasts generally covered zero, and no consistent trends of platform usage moderation • Low usage and short follow-up periods may bias effect estimation • A precise, intuitive, and objective measure of user engagement can capture different eHealth use patterns	• Extend follow-up to 6 months • Use appropriate models to account for non-usage: treat usage as binary moderator (high vs. low) or as continuous predictor of outcomes

*Notes:* DDEP: Digital Dyadic Empowerment Program; Int.: Intervention; HPLP-IICR: Health Promoting Lifestyle Profile-II Chinese version Revised; DAS-7: seven-item Dyadic Adjustment Scale; AE: adverse event; SO: significant other; BP: blood pressure; eGFR: estimated Glomerular Filtration Rate; CI: confidence interval.

## 6 Conclusion

The “*Kidney Lifestyle*” dyadic digital health intervention is safe and feasible to trial within a routine clinical care setting for patients with chronic kidney disease and their significant others. This study achieved high rates of recruitment and retention, providing a strong foundation for future research. It also generated critical insights into user engagement patterns, leading to a clear, actionable roadmap for a definitive trial. The findings provide optimism that this digitally-enabled, family-supported model of care can be rigorously evaluated for its effectiveness in improving chronic kidney disease care.

## Declaration of generative AI and AI-assisted technologies in the manuscript preparation process

OpenAI’s ChatGPT (version 5.2) and Google Gemini Pro (version 3) were used to improve the language clarity and structural coherence of this manuscript. All text revisions were reviewed, verified, and approved by the authors, who take full responsibility for the final content.

## Funding

This study was supported by the National Health Research Institutes (NHRI), Taiwan, through the Integrated Research Grants in Health and Medical Sciences (Innovative Research Grant, Grant No. NHRI-EX113–11106PI). The funding agency had no role in the study design; data collection, management, analysis, or interpretation; or the preparation, review, or approval of the manuscript.

## CRediT authorship contribution statement

**Chun-Yi Ho:** Writing – review & editing, Writing – original draft, Project administration, Methodology, Investigation, Formal analysis. **Deborah Siregar:** Writing – review & editing, Writing – original draft, Project administration. **Wei-Hung Lin:** Writing – review & editing, Investigation. **Junne-Ming Sung:** Writing – review & editing, Investigation. **Ming-Cheng Wang:** Writing – review & editing, Investigation. **Miaofen Yen:** Writing – review & editing, Supervision, Methodology, Funding acquisition, Conceptualization.

## Declaration of competing interest

The authors declare the following financial interests/personal relationships which may be considered as potential competing interests:

Miaofen Yen reports financial support was provided by National Health Research Institutes. If there are other authors, they declare that they have no known competing financial interests or personal relationships that could have appeared to influence the work reported in this paper.

## Appendix Supplementary materials

Image, application 1
