# Supplementary material for: Dyadic mobile intervention empowering lifestyle modification in chronic kidney disease management: A feasibility randomized controlled trial
Source: Int J Nurs Stud Adv. 2026 May 14;10:100549. doi: 10.1016/j.ijnsa.2026.100549 (PMC13202244; doi:10.1016/j.ijnsa.2026.100549)
Supplement: Supplementary file 1 [file mmc1.docx]

**Supplementary Appendix**

**Secondary Clinical Outcomes: Measures, Analytic Methods, and Results**

**Manuscript Title:**

Dyadic mobile intervention empowering lifestyle modification in chronic kidney disease management: A feasibility randomized controlled trial

**Authors:**

Chun-Yi **Ho**^1^, Deborah **Siregar**^1^, Wei-Hung **Lin**^2^, Junne-Ming **Sung**^2^, Ming-Cheng **Wang**^2^, Miaofen **Yen**^1,^ *.

**Affiliations:**

1. Department of Nursing, College of Medicine, National Cheng Kung University, Tainan City, Taiwan
2. Department of Internal Medicine, National Cheng Kung University Hospital, College of Medicine, National Cheng Kung University, Tainan City, Taiwan

**Corresponding author**:

*Miaofen Yen, PhD, RN, FAAN

Department of Nursing, College of Medicine, National Cheng Kung University

No. 1, Daxue Rd., East Dist., Tainan City 701401, Taiwan

phone: 886 6 2353535 ext. 5823

email: [miaofen@mail.ncku.edu.tw](mailto:miaofen@mail.ncku.edu.tw)

# Part 1. Measures and Data Collection Procedures

## *Measures*

Secondary clinical outcomes of this feasibility study included:

1. **Kidney Function Indicators:** Research assistants, authorized by physicians, retrieved patients’ biochemical reports from the electronic medical record system. The estimated Glomerular Filtration Rate (eGFR), calculated using the Isotope Dilution Mass Spectrometry - Modification of Diet in Renal Disease (IDMS-MDRD) formula, and the corresponding chronic kidney disease (CKD) stage were obtained.
2. **Stages of Change for Lifestyle Modification:** Each dyad’s perceived motivation for lifestyle modification was assessed using five descriptive statements corresponding to different stages of change based on the Transtheoretical Model (TTM). These stages included: *precontemplation*, *contemplation*, *preparation*, *action*, and *maintenance*. Similar assessment methods have been applied in and Chao et al. (2022a).
3. **Health-Promoting Lifestyle Profile-II (HPLP-II):** Patients’ lifestyle was assessed using the validated Chinese version of the Health-Promoting Lifestyle Profile-II (HPLP-II) questionnaire (Teng et al., 2010). This scale contains 52 items scored on a 4-point Likert scale, with scores ranging from 52 to 208. It includes six subscales: *Health Responsibility*, *Physical Activity*, *Nutrition*, *Spiritual Growth*, *Interpersonal Relations*, and *Stress Management*. Higher scores indicate healthier lifestyles.
4. **Adherence to Healthy Behaviors Scale (AHBS):** The validated Adherence to Healthy Behaviors Scale (AHBS), developed by Huang et al. (2021), was used to evaluate patients’ adherence to healthy behaviors over the past month. This scale consists of 13 items, scored on a 5-point Likert scale, with scores ranging from 0 to 52. It encompasses three subscales: *Abstinence*, *Recommendations*, and *Precautions*. Higher scores indicate better adherence to healthy behaviors.
5. **Helping Relationships from Significant Others (HRSO):** The validated Chinese version of the Helping Relationships from Significant Others Scale (HRSO), developed and validated by Chao et al. (2022b), was used to measure patients’ perceived support by significant others regarding disease or health-related matters within the past month. This scale comprises 15 items, scored on a 7-point Likert scale, with scores ranging from 15 to 105. It includes three subscales: *Understanding*, *Caring*, and *Coaching*. Higher scores indicate greater support from significant others.
6. **Dyadic Adjustment Scale-7 (DAS-7):** The seven-item Dyadic Adjustment Scale (DAS-7), developed and validated by Hunsley et al. (2001), was used to measure both patients’ and significant others’ self-assessed relationship quality within each dyad. This scale includes seven items; the first six items are scored on a 6-point scale, and the seventh item is scored on a 7-point scale, with total scores ranging from 0 to 36. Higher scores indicate better dyadic adjustment.
7. **Quality of Life (WHOQOL-BREF):** Both patients’ and significant others’ quality of life (QoL) over the past two weeks was evaluated using the validated Taiwanese version of the World Health Organization Quality of Life-BREF (WHOQOL-BREF) questionnaire (Yao et al., 2002). This scale consists of 28 items scored on a 5-point Likert scale, including two global items (general QoL and general Health) and four subscales: *Physical*, *Psychological*, *Social*, and *Environment* domains. Scores for each domain range from 4 to 20, with higher scores indicating better QoL.

## *Data Collection Procedures*

These questionnaire-based clinical indicators were collected by research assistants through paper-based forms at outpatient visits at two time points: baseline (T0, when CKD dyads consented to participate in the study) and approximately three months later at follow-up (T1). Kidney function indicators were recorded from the biochemical test results closest to these two time points. Participants unable to complete the questionnaires onsite were asked to take them home, complete within one week, and submit at the next visit (participants in the intervention group could also submit completed questionnaires by taking photos via the digital platform). If questionnaires were returned incomplete, participants were requested to complete any missing items. Unexpected circumstances, such as lost questionnaires, forgotten completion, or completed but forgotten questionnaires, were addressed by providing replacement paper forms onsite or through digital platform follow-up.

# Part 2. Statistical Approach and Model Specifications

This study adopted an intention-to-treat (ITT) principle for analyzing all study participants. A modified intention-to-treat (mITT) analysis, excluding participants who dropped out, was performed as necessary. In addition, per-protocol (PP) analyses were conducted to explore deviations from the ITT principle and their impact on effect estimates, compared to mITT results. Specifically, intervention group participants were further categorized by high and low adherence based on the 90-day platform usage threshold of 50%, generating redefined analytical groups (high-usage, low-usage, control). Analyses were conducted using IBM SPSS Statistics (version 25.0).

Given the feasibility study objectives were not to compare treatment efficacy, statistical significance was not reported or interpreted. The methods for each variable type were as follows:

1. Ordinal Variables: For CKD stage and TTM stage of change, we summarized transitions from baseline (T0) to follow-up (T1) using three ordered categories (CKD: *recover*, *stable*, *progress*; TTM: *regress*, *maintain*, *advance*). For effect estimation, we dichotomized outcomes into favorable vs unfavorable (CKD: *recover*/*stable* vs *progress*; TTM: *regress* vs *maintain*/*advance*) and reported odds ratios (ORs) with 95% CIs, comparing intervention vs control in the mITT analysis and high-usage vs control in the PP analysis.
2. Continuous Variables: Generalized Linear Mixed Models (GLMM) provided estimates, standard errors, *t*-values, and 95% CIs for pre-post score differences between groups. Depending on data source, GLMM analyses were categorized into two types:
   1. Patient-only continuous variables: These included eGFR, HPLP-II, AHBS, and HRSO scores. Models used a normal distribution and identity link function. Fixed effects included the main effect of measurement time (T0, T1) and the interaction of group × measurement time. Random effects incorporated intercept and measurement time with variance component covariance structure to estimate individual participant variation.
   2. Dyadic continuous variables: These included quality of life (WHOQOL-BREF) and dyadic adjustment (DAS-7) scores. Models used a normal distribution and identity link function. Fixed effects included main effects of participant identity (patient, significant other), measurement time, and interaction of group × measurement time. Random effects incorporated intercept and measurement time with variance component and compound symmetry covariance structures to estimate variations between dyads and between individuals within dyads, respectively.

Both analyses employed the Satterthwaite approximation to ensure accurate degrees-of-freedom estimates despite unbalanced data across groups. Robust estimation adjusted standard errors of fixed effects to address model assumption violations. Missing data were handled via listwise deletion without imputation.

Ordinal clinical outcomes were reported using frequencies and percentages. Continuous clinical outcomes were reported numerically and visualized through pre-post change graphs for each analytical group × measurement time to illustrate trends clearly.

# Part 3. Results (Summary Tables and Figures)

**Table S1**

*Pre-Post Changes in CKD Stage and TTM Stage of Change*

*mITT Analysis (excluding participants who withdrew)*

| Variable | All  (*N* = 55) | Intervention  (DDEP + UC)  (*n* = 26) | Control  (UC only)  (*n* = 29) | Odds Ratio [95% CI] ^b^  Intervention vs Control |
| --- | --- | --- | --- | --- |
| **CKD Stage Change** ^a^ |  |  |  | 0.69  [0.18, 2.62] |
| Recover | 6 (10.9) | 2 (7.7) | 4 (13.8) |  |
| Stable | 38 (69.1) | 18 (69.2) | 20 (69.0) |  |
| Progress | 11 (20.0) | 6 (23.1) | 5 (17.2) |  |
| **TTM Stage Change** ^a^ |  |  |  | 1.15  [0.27, 4.82] |
| Regress | 9 (16.4) | 4 (15.4) | 5 (17.2) |  |
| Maintain | 33 (60.0) | 14 (53.8) | 19 (65.5) |  |
| Advance | 13 (23.6) | 8 (30.8) | 5 (17.2) |  |

*PP Analysis (intervention group subdivided by platform engagement level)*

| Variable | Low Usage  (*n* = 12) | High Usage  (*n* = 14) | Control  (UC only)  (*n* = 29) | Odds Ratio [95% CI] ^b^  High usage vs Control |
| --- | --- | --- | --- | --- |
| **CKD Stage Change** ^a^ |  |  |  | 0.76  [0.15, 3.78] |
| Recover | 0 (0.0) | 2 (14.3) | 4 (13.8) |  |
| Stable | 9 (75.0) | 9 (64.3) | 20 (69.0) |  |
| Progress | 3 (25.0) | 3 (21.4) | 5 (17.2) |  |
| **TTM Stage Change** ^a^ |  |  |  | 0.76  [0.15, 3.78] |
| Regress | 1 (8.3) | 3 (21.4) | 5 (17.2) |  |
| Maintain | 7 (58.3) | 7 (50.0) | 19 (65.5) |  |
| Advance | 4 (33.3) | 4 (28.6) | 5 (17.2) |  |

*Note.* ^a^ Number (%); ^b^ Odds ratios compare the odds of being in the favorable categories (CKD: Recover or Stable; TTM: Maintain or Advance) vs unfavorable (CKD: Progress; TTM: Regress) between the groups indicated. CKD = chronic kidney disease; TTM = Transtheoretical Model; mITT = modified intention-to-treat; DDEP = Digital Dyadic Empowerment Program; UC = usual care; CI = confidence interval; PP = per protocol.

**Table S2**

*GLMM Summary of eGFR (Estimated Glomerular Filtration Rate)*

*Fixed Effects Model Test Overview*

| mITT Population (*N* = 55) | | PP Population (*N* = 55) | |
| --- | --- | --- | --- |
| Source | *F* (df1, df2) | Source | *F* (df1, df2) |
| Corrected Model | 2.26 (3, 106) | Corrected Model | 5.38 (5, 104) |
| Timepoint | 4.72 (1, 106) | Timepoint | 6.44 (1, 104) |
| Group × Timepoint | 0.63 (2, 106) | Group × Timepoint | 1.20 (4, 104) |

*Estimated Mean Differences (Post - Pre) by Group*

| Group (*n*) | Difference  (T1 - T0) | Std. Error | *t* (df) | 95% CI | |
| --- | --- | --- | --- | --- | --- |
|  |  |  |  | Lower | Upper |
| Control (29) | -4.12 (41.30 - 45.43) | 2.51 | -1.64 (106) | -9.11 | 0.86 |
| Intervention (26) | -1.71 (44.59 - 46.29) | 0.95 | -1.81 (106) | -3.58 | 0.17 |
| Low Usage (12) | -3.28 (39.52 - 42.80) | 0.94 | -3.48 (104) | -5.14 | -1.41 |
| High Usage (14) | -0.36 (48.92 - 49.29) | 1.47 | -0.25 (104) | -3.27 | 2.54 |

*Estimated Differences Visualized Across Timepoints by Group*

**mITT Population** **PP Population**


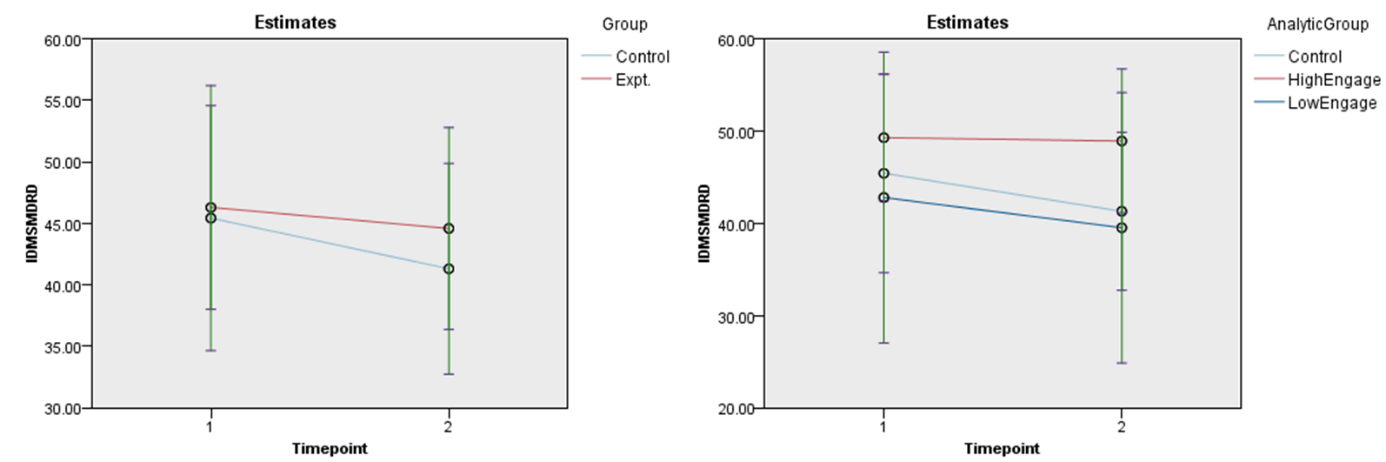


*Note.* GLMM = generalized linear mixed model; mITT = modified intention-to-treat (excluding withdrawals); PP = per protocol (intervention group further categorized by platform usage level: high vs. low).

**Table S3**

*GLMM Summary of Health-Promoting Lifestyle Profile-II (HPLP-II)*

*Fixed Effects Model Test Overview*

| mITT Population (*N* = 55) | | PP Population (*N* = 55) | |
| --- | --- | --- | --- |
| Source | *F* (df1, df2) | Source | *F* (df1, df2) |
| Corrected Model | 2.69 (3, 28) | Corrected Model | 2.05 (5, 16) |
| Timepoint | 0.13 (1, 44) | Timepoint | 0.23 (1, 18) |
| Group × Timepoint | 3.53 (2, 47) | Group × Timepoint | 2.45 (4, 21) |

*Estimated Mean Differences (Post - Pre) by Group*

| Group (*n*) | Difference  (T1 - T0) | Std. Error | *t* (df) | 95% CI | |
| --- | --- | --- | --- | --- | --- |
|  |  |  |  | Lower | Upper |
| Control (29) | -8.17  (132.52 - 140.69) | 5.22 | -1.57 (106) | -18.52 | 2.18 |
| Intervention (26) | 5.96  (148.15 - 142.19) | 3.02 | 1.98 (10) | -0.77 | 12.70 |
| Low Usage (12) | 6.25  (141.58 - 135.33) | 3.96 | 1.58 (6) | -3.48 | 15.98 |
| High Usage (14) | 5.71  (153.79 - 148.07) | 4.45 | 1.28 (13) | -3.92 | 15.35 |

*Estimated Differences Visualized Across Timepoints by Group*

**mITT Population** **PP Population**


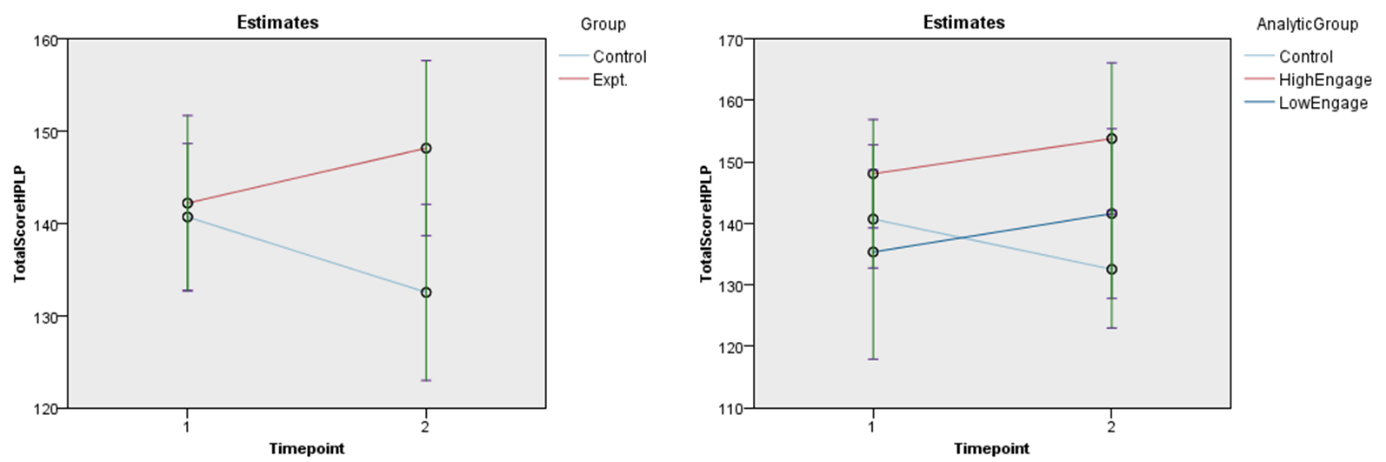


*Note.* GLMM = generalized linear mixed model; mITT = modified intention-to-treat (excluding withdrawals); PP = per protocol (intervention group further categorized by platform usage level: high vs. low).

**Table S4**

*GLMM Summary of HPLP-II Health Responsibility*

*Fixed Effects Model Test Overview*

| mITT Population (*N* = 55) | | PP Population (*N* = 55) | |
| --- | --- | --- | --- |
| Source | *F* (df1, df2) | Source | *F* (df1, df2) |
| Corrected Model | 1.69 (3, 41) | Corrected Model | 1.31 (5, 5) |
| Timepoint | 0.07 (1, 46) | Timepoint | 0.25 (1, 33) |
| Group × Timepoint | 2.48 (2, 48) | Group × Timepoint | 1.38 (4, 26) |

*Estimated Mean Differences (Post - Pre) by Group*

| Group (*n*) | Difference  (T1 - T0) | Std. Error | *t* (df) | 95% CI | |
| --- | --- | --- | --- | --- | --- |
|  |  |  |  | Lower | Upper |
| Control (29) | -1.79  (24.21 - 26.00) | 1.15 | -1.56 (82) | -4.08 | 0.50 |
| Intervention (26) | 1.42  (25.65 - 24.23) | 0.88 | 1.63 (22) | -0.39 | 3.24 |
| Low Usage (12) | 1.67  (25.50 - 23.83) | 1.71 | 0.98 (64) | -1.74 | 5.07 |
| High Usage (14) | 1.21  (25.79 - 24.57) | 0.71 | 1.71 (3) | -1.25 | 3.67 |

*Estimated Differences Visualized Across Timepoints by Group*

**mITT Population** **PP Population**


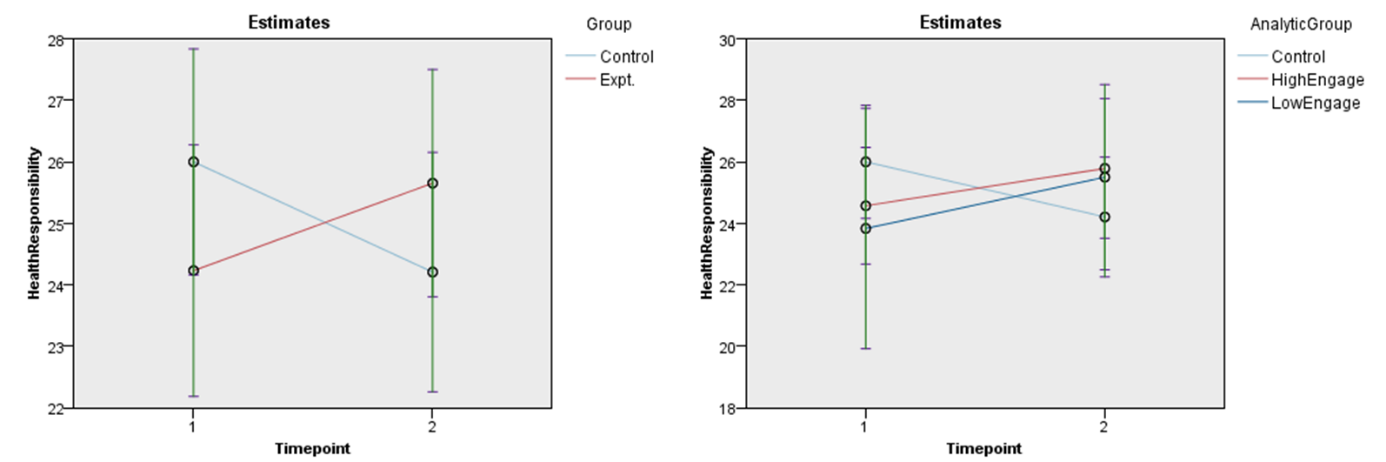


*Note.* GLMM = generalized linear mixed model; mITT = modified intention-to-treat (excluding withdrawals); PP = per protocol (intervention group further categorized by platform usage level: high vs. low).

**Table S5**

*GLMM Summary of HPLP-II Physical Activity*

*Fixed Effects Model Test Overview*

| mITT Population (*N* = 55) | | PP Population (*N* = 55) | |
| --- | --- | --- | --- |
| Source | *F* (df1, df2) | Source | *F* (df1, df2) |
| Corrected Model | 2.14 (3, 44) | Corrected Model | 1.41 (5, 38) |
| Timepoint | 0.10 (1, 47) | Timepoint | 1.17 (1, 31) |
| Group × Timepoint | 2.89 (2, 48) | Group × Timepoint | 1.64 (4, 37) |

*Estimated Mean Differences (Post - Pre) by Group*

| Group (*n*) | Difference  (T1 - T0) | Std. Error | *t* (df) | 95% CI | |
| --- | --- | --- | --- | --- | --- |
|  |  |  |  | Lower | Upper |
| Control (29) | -1.28  (18.97 - 20.24) | 0.94 | -1.36 (74) | -3.15 | 0.60 |
| Intervention (26) | 1.65  (20.73 - 19.08) | 0.78 | 2.13 (27) | 0.07 | 3.24 |
| Low Usage (12) | 1.00  (19.50 - 18.50) | 1.07 | 0.94 (20) | -1.23 | 3.23 |
| High Usage (14) | 2.21  (21.79 - 19.57) | 1.09 | 2.03 (30) | -0.01 | 4.44 |

*Estimated Differences Visualized Across Timepoints by Group*

**mITT Population** **PP Population**


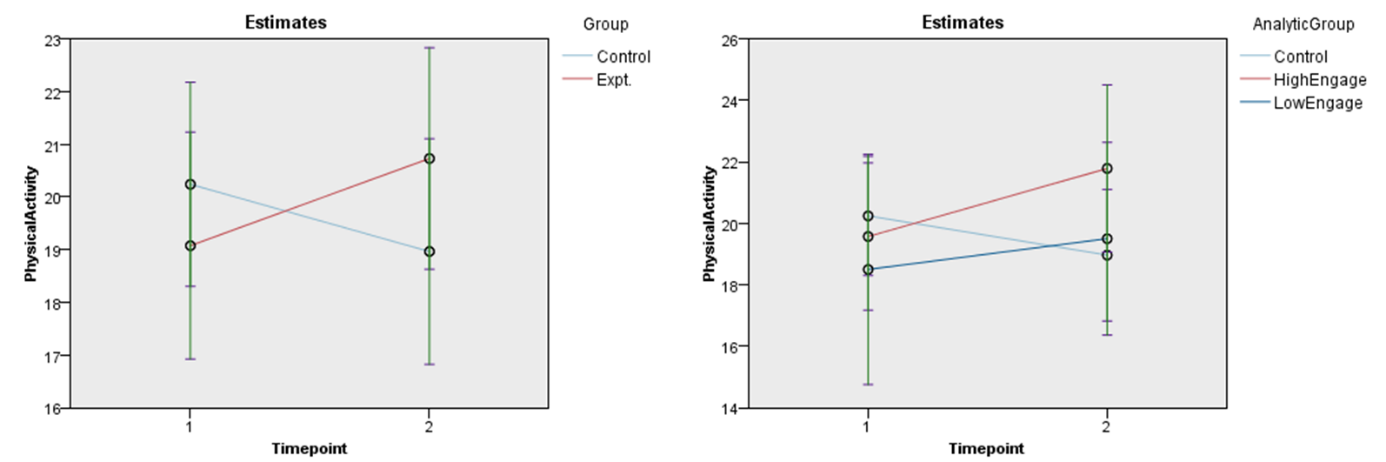


*Note.* GLMM = generalized linear mixed model; mITT = modified intention-to-treat (excluding withdrawals); PP = per protocol (intervention group further categorized by platform usage level: high vs. low).

**Table S6**

*GLMM Summary of HPLP-II Nutrition*

*Fixed Effects Model Test Overview*

| mITT Population (*N* = 55) | | PP Population (*N* = 55) | |
| --- | --- | --- | --- |
| Source | *F* (df1, df2) | Source | *F* (df1, df2) |
| Corrected Model | 0.60 (3, 44) | Corrected Model | 1.13 (5, 43) |
| Timepoint | 0.05 (1, 47) | Timepoint | 0.23 (1, 30) |
| Group × Timepoint | 0.84 (2, 49) | Group × Timepoint | 1.41 (4, 43) |

*Estimated Mean Differences (Post - Pre) by Group*

| Group (*n*) | Difference  (T1 - T0) | Std. Error | *t* (df) | 95% CI | |
| --- | --- | --- | --- | --- | --- |
|  |  |  |  | Lower | Upper |
| Control (29) | -0.21  (24.03 - 24.24) | 0.76 | -0.27 (75) | -1.72 | 1.31 |
| Intervention (26) | 0.42  (25.50 - 25.08) | 0.62 | 0.68 (27) | -0.85 | 1.69 |
| Low Usage (12) | 0.75  (24.25 - 23.50) | 0.83 | 0.91 (17) | -0.99 | 2.49 |
| High Usage (14) | 0.14  (26.57 - 26.43) | 0.90 | 0.16 (33) | -1.68 | 1.97 |

*Estimated Differences Visualized Across Timepoints by Group*

**mITT Population** **PP Population**


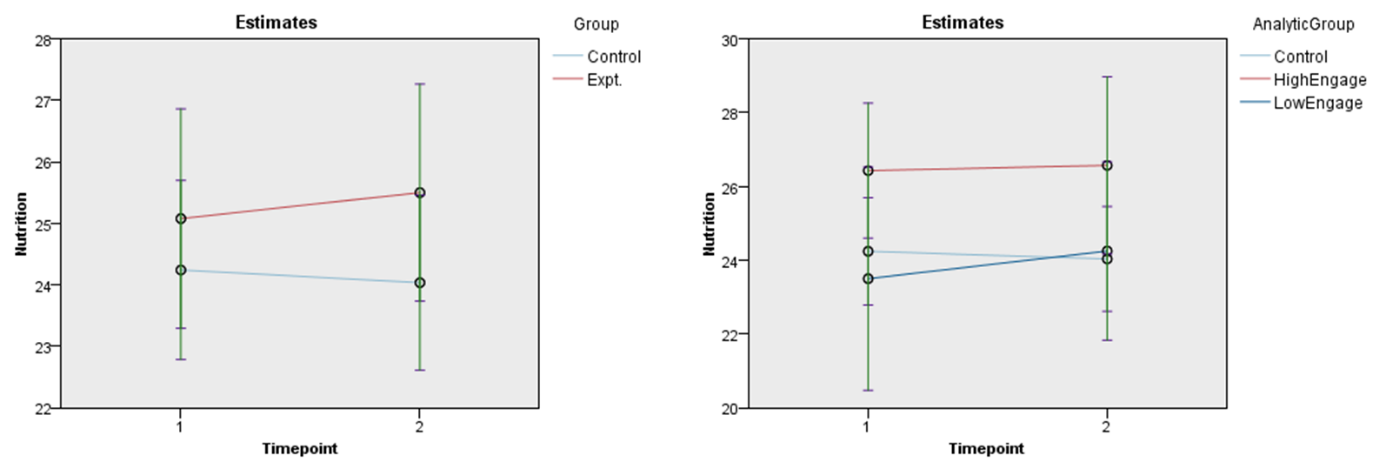


*Note.* GLMM = generalized linear mixed model; mITT = modified intention-to-treat (excluding withdrawals); PP = per protocol (intervention group further categorized by platform usage level: high vs. low).

**Table S7**

*GLMM Summary of HPLP-II Spiritual Growth*

*Fixed Effects Model Test Overview*

| mITT Population (*N* = 55) | | PP Population (*N* = 55) | |
| --- | --- | --- | --- |
| Source | *F* (df1, df2) | Source | *F* (df1, df2) |
| Corrected Model | 2.52 (3, 20) | Corrected Model | 3.10 (5, 11) |
| Timepoint | 1.49 (1, 43) | Timepoint | 0.43 (1, 15) |
| Group × Timepoint | 3.78 (2, 47) | Group × Timepoint | 3.87 (4, 17) |

*Estimated Mean Differences (Post - Pre) by Group*

| Group (*n*) | Difference  (T1 - T0) | Std. Error | *t* (df) | 95% CI | |
| --- | --- | --- | --- | --- | --- |
|  |  |  |  | Lower | Upper |
| Control (29) | -2.28  (22.83 - 25.10) | 1.30 | -1.75 (106) | -4.86 | 0.31 |
| Intervention (26) | 0.50  (26.62 - 26.12) | 0.65 | 0.77 (6) | -1.08 | 2.08 |
| Low Usage (12) | 1.08  (24.75 - 23.67) | 0.78 | 1.39 (3) | -1.66 | 3.83 |
| High Usage (14) | 0.00  (28.21 - 28.21) | 0.99 | 0.00 (9) | -2.24 | 2.24 |

*Estimated Differences Visualized Across Timepoints by Group*

**mITT Population** **PP Population**


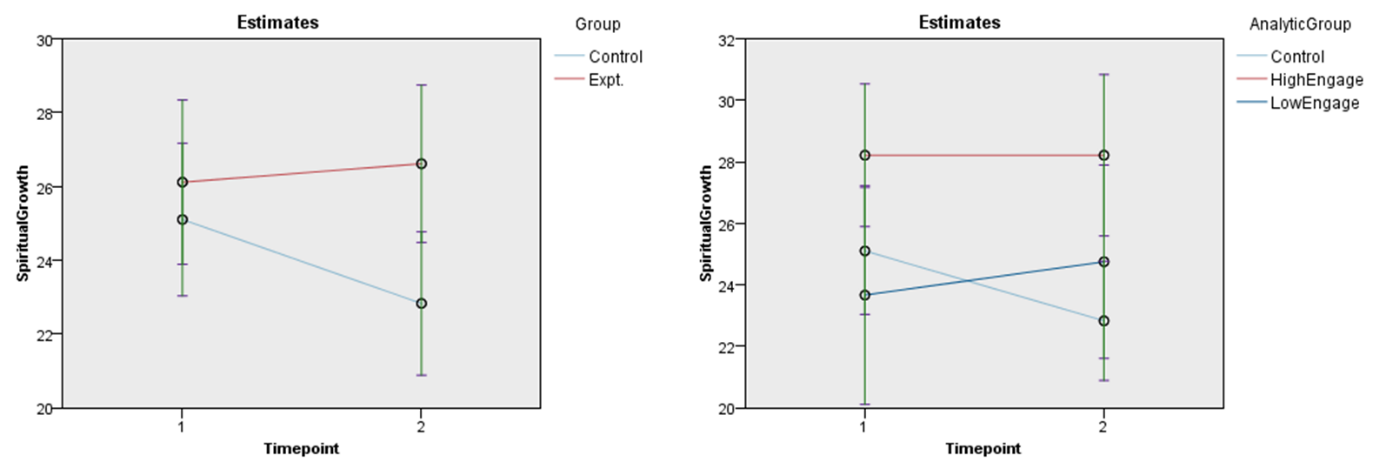


*Note.* GLMM = generalized linear mixed model; mITT = modified intention-to-treat (excluding withdrawals); PP = per protocol (intervention group further categorized by platform usage level: high vs. low).

**Table S8**

*GLMM Summary of HPLP-II Interpersonal Relations*

*Fixed Effects Model Test Overview*

| mITT Population (*N* = 55) | | PP Population (*N* = 55) | |
| --- | --- | --- | --- |
| Source | *F* (df1, df2) | Source | *F* (df1, df2) |
| Corrected Model | 4.31 (3, 26) | Corrected Model | 3.08 (5, 11) |
| Timepoint | 0.39 (1, 44) | Timepoint | 1.87 (1, 18) |
| Group × Timepoint | 4.88 (2, 46) | Group × Timepoint | 3.32 (4, 22) |

*Estimated Mean Differences (Post - Pre) by Group*

| Group (*n*) | Difference  (T1 - T0) | Std. Error | *t* (df) | 95% CI | |
| --- | --- | --- | --- | --- | --- |
|  |  |  |  | Lower | Upper |
| Control (29) | -0.62  (22.83 - 23.45) | 1.13 | -0.55 (106) | -2.85 | 1.61 |
| Intervention (26) | 1.42  (26.96 - 25.54) | 0.63 | 2.27 (9) | 0.00 | 2.85 |
| Low Usage (12) | 2.00  (26.42 - 24.42) | 0.99 | 2.03 (11) | -0.17 | 4.17 |
| High Usage (14) | 0.93  (27.43 - 26.50) | 0.78 | 1.19 (6) | -0.99 | 2.85 |

*Estimated Differences Visualized Across Timepoints by Group*

**mITT Population** **PP Population**


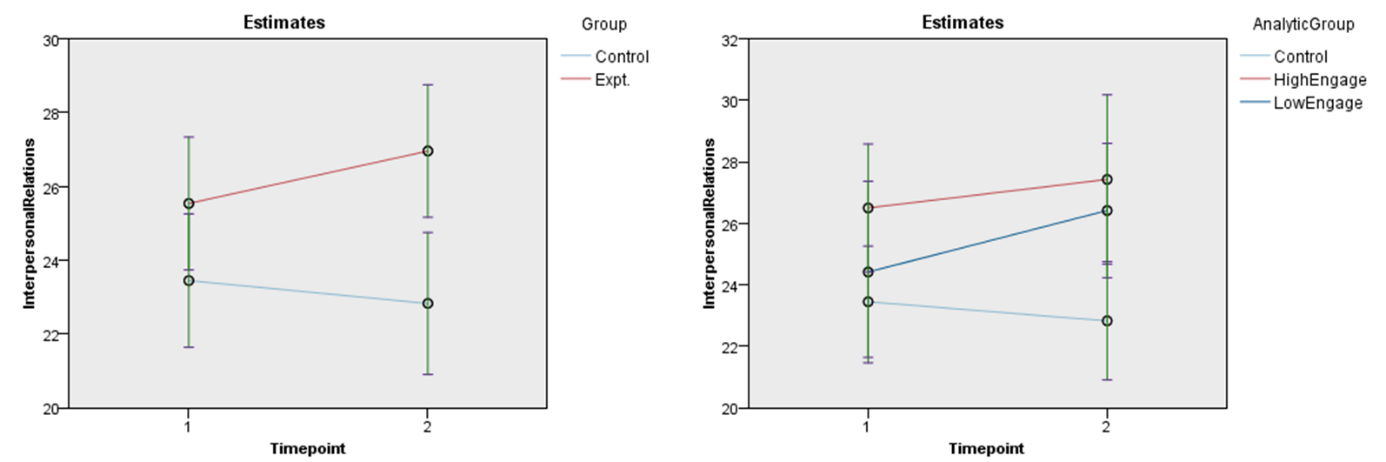


*Note.* GLMM = generalized linear mixed model; mITT = modified intention-to-treat (excluding withdrawals); PP = per protocol (intervention group further categorized by platform usage level: high vs. low).

**Table S9**

*GLMM Summary of HPLP-II Stress Management*

*Fixed Effects Model Test Overview*

| mITT Population (*N* = 55) | | PP Population (*N* = 55) | |
| --- | --- | --- | --- |
| Source | *F* (df1, df2) | Source | *F* (df1, df2) |
| Corrected Model | 2.60 (3, 29) | Corrected Model | 2.24 (5, 5) |
| Timepoint | 1.72 (1, 44) | Timepoint | 0.53 (1, 17) |
| Group × Timepoint | 3.80 (2, 46) | Group × Timepoint | 2.61 (4, 14) |

*Estimated Mean Differences (Post - Pre) by Group*

| Group (*n*) | Difference  (T1 - T0) | Std. Error | *t* (df) | 95% CI | |
| --- | --- | --- | --- | --- | --- |
|  |  |  |  | Lower | Upper |
| Control (29) | -2.00  (19.66 - 21.66) | 0.96 | -2.09 (106) | -3.90 | -0.10 |
| Intervention (26) | 0.54  (22.69 - 22.15) | 0.57 | 0.95 (11) | -0.72 | 1.79 |
| Low Usage (12) | -0.25  (21.17 - 21.42) | 0.47 | -0.53 (1) | -5.46 | 4.96 |
| High Usage (14) | 1.21  (24.00 - 22.79) | 0.94 | 1.30 (22) | -0.73 | 3.16 |

*Estimated Differences Visualized Across Timepoints by Group*

**mITT Population** **PP Population**


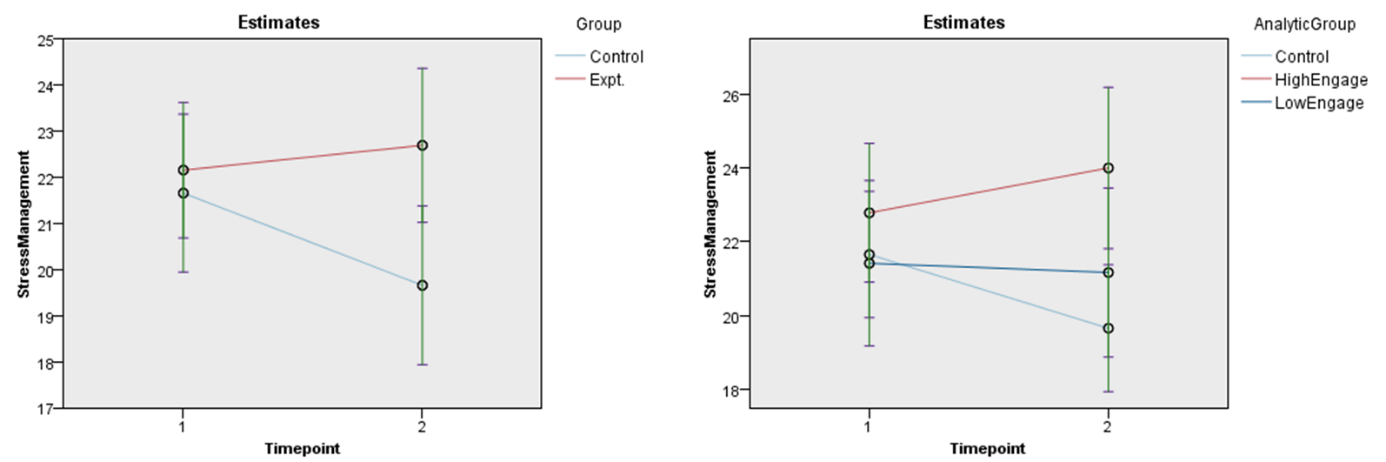


*Note.* GLMM = generalized linear mixed model; mITT = modified intention-to-treat (excluding withdrawals); PP = per protocol (intervention group further categorized by platform usage level: high vs. low).

**Table S10**

*GLMM Summary of Adherence to Healthy Behaviors Scale (AHBS)*

*Fixed Effects Model Test Overview*

| mITT Population (*N* = 55) | | PP Population (*N* = 55) | |
| --- | --- | --- | --- |
| Source | *F* (df1, df2) | Source | *F* (df1, df2) |
| Corrected Model | 2.84 (3, 28) | Corrected Model | 3.34 (5, 10) |
| Timepoint | 1.76 (1, 44) | Timepoint | 3.41 (1, 22) |
| Group × Timepoint | 1.11 (2, 47) | Group × Timepoint | 1.61 (4, 22) |

*Estimated Mean Differences (Post - Pre) by Group*

| Group (*n*) | Difference  (T1 - T0) | Std. Error | *t* (df) | 95% CI | |
| --- | --- | --- | --- | --- | --- |
|  |  |  |  | Lower | Upper |
| Control (29) | 0.10 (45.07 - 44.97) | 1.23 | 0.08 (105) | -2.33 | 2.54 |
| Intervention (26) | 1.81 (47.04 - 45.23) | 0.75 | 2.41 (12) | 0.17 | 3.45 |
| Low Usage (12) | 1.08 (45.58 - 44.50) | 1.30 | 0.83 (22) | -1.62 | 3.79 |
| High Usage (14) | 2.43 (48.29 - 45.86) | 0.79 | 3.06 (4) | 0.24 | 4.62 |

*Estimated Differences Visualized Across Timepoints by Group*

**mITT Population** **PP Population**


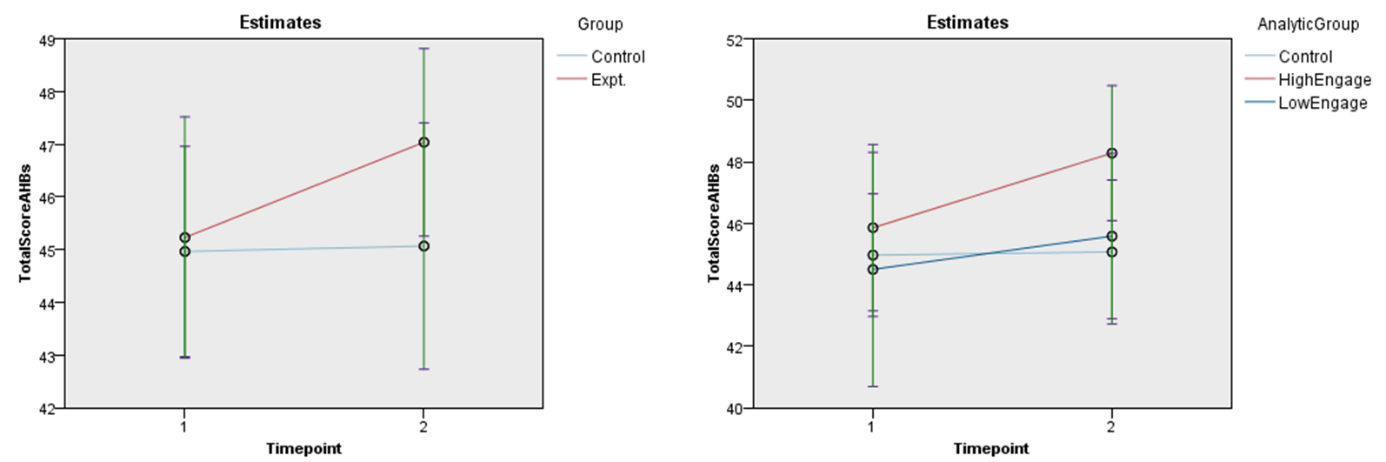


*Note.* GLMM = generalized linear mixed model; mITT = modified intention-to-treat (excluding withdrawals); PP = per protocol (intervention group further categorized by platform usage level: high vs. low).

**Table S11**

*GLMM Summary of AHBS Abstinence*

*Fixed Effects Model Test Overview*

| mITT Population (*N* = 55) | | PP Population (*N* = 55) | |
| --- | --- | --- | --- |
| Source | *F* (df1, df2) | Source | *F* (df1, df2) |
| Corrected Model | 2.37 (3, 106) | Corrected Model | 1.74 (5, 104) |
| Timepoint | 5.32 (1, 41) | Timepoint | 5.91 (1, 104) |
| Group × Timepoint | 1.05 (2, 25) | Group × Timepoint | 1.70 (4, 104) |

*Estimated Mean Differences (Post - Pre) by Group*

| Group (*n*) | Difference  (T1 - T0) | Std. Error | *t* (df) | | 95% CI | | |
| --- | --- | --- | --- | --- | --- | --- | --- |
|  |  |  |  |  | Lower | | Upper |
| Control (29) | 1.45 (14.90 - 13.45) | 0.76 | | 1.92 (106) | | -0.05 | 2.95 |
| Intervention (26) | 0.39 (15.15 - 14.77) | 0.25 | | 1.54 (1) | | -1.21 | 1.98 |
| Low Usage (12) | 0.33 (15.58 - 15.25) | 0.40 | | 0.84 (104) | | -0.45 | 1.12 |
| High Usage (14) | 0.43 (14.79 - 14.36) | 0.32 | | 1.36 (104) | | -0.20 | 1.05 |

*Estimated Differences Visualized Across Timepoints by Group*

**mITT Population** **PP Population**


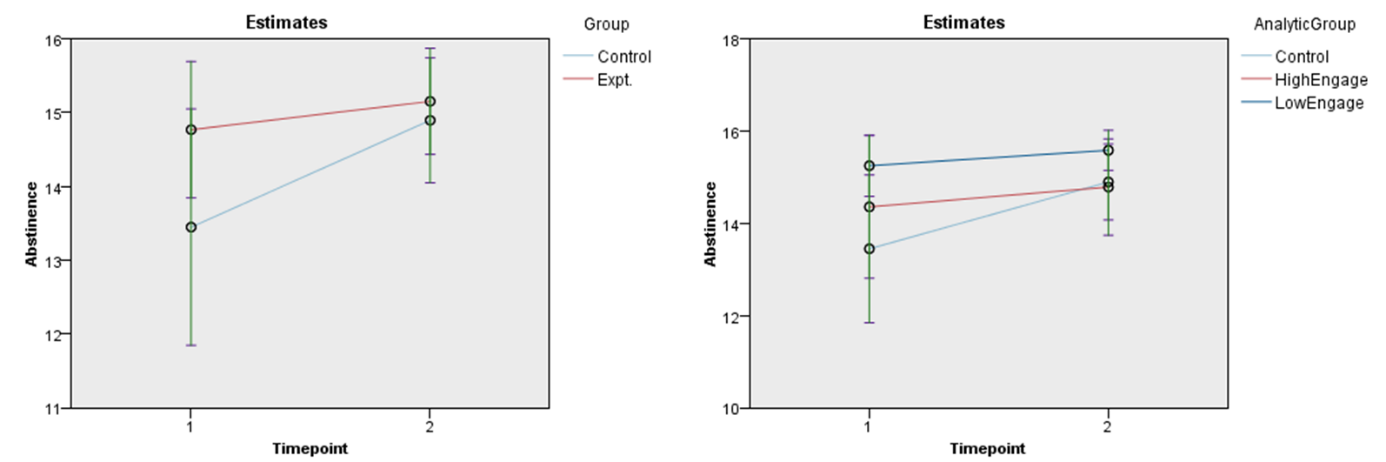


*Note.* GLMM = generalized linear mixed model; mITT = modified intention-to-treat (excluding withdrawals); PP = per protocol (intervention group further categorized by platform usage level: high vs. low).

**Table S12**

*GLMM Summary of AHBS Recommendations*

*Fixed Effects Model Test Overview*

| mITT Population (*N* = 55) | | PP Population (*N* = 55) | |
| --- | --- | --- | --- |
| Source | *F* (df1, df2) | Source | *F* (df1, df2) |
| Corrected Model | 1.40 (3, 46) | Corrected Model | 1.98 (5, 27) |
| Timepoint | 0.02 (1, 48) | Timepoint | 0.17 (1, 41) |
| Group × Timepoint | 2.09 (2, 48) | Group × Timepoint | 2.29 (4, 31) |

*Estimated Mean Differences (Post - Pre) by Group*

| Group (*n*) | Difference  (T1 - T0) | Std. Error | *t* (df) | | 95% CI | | |
| --- | --- | --- | --- | --- | --- | --- | --- |
|  |  |  |  |  | Lower | | Upper |
| Control (29) | -0.76  (20.03 - 20.79) | 0.57 | | -1.34 (64) | | -1.89 | 0.37 |
| Intervention (26) | 0.65  (21.39 - 20.73) | 0.52 | | 1.27 (35) | | -0.39 | 1.70 |
| Low Usage (12) | 0.25  (20.58 - 20.33) | 0.93 | | 0.27 (76) | | -1.60 | 2.10 |
| High Usage (14) | 1.00  (22.07 - 21.07) | 0.52 | | 1.94 (10) | | -0.15 | 2.15 |

*Estimated Differences Visualized Across Timepoints by Group*

**mITT Population** **PP Population**


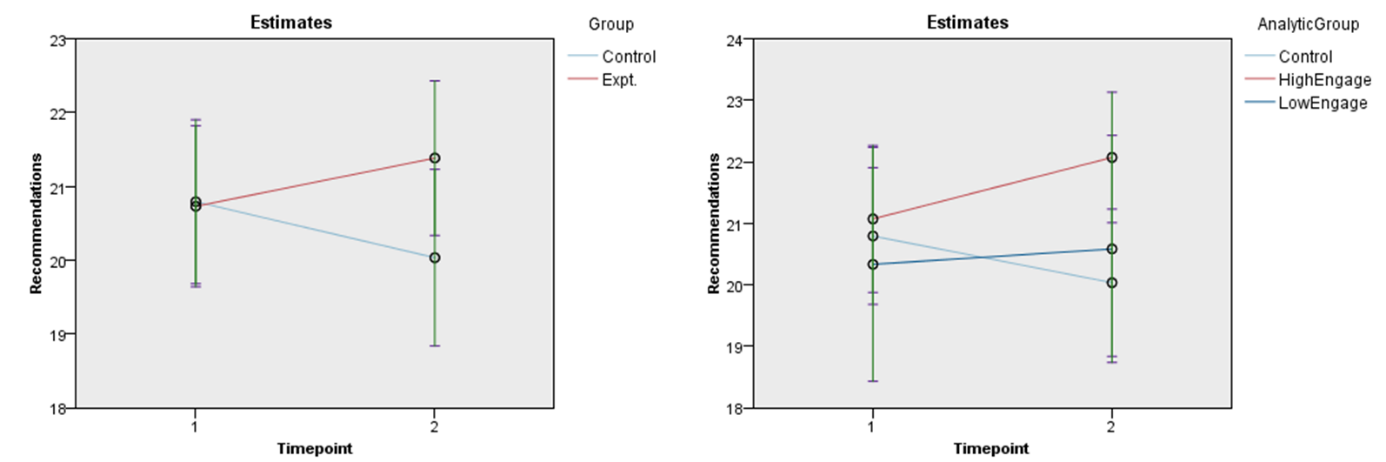


*Note.* GLMM = generalized linear mixed model; mITT = modified intention-to-treat (excluding withdrawals); PP = per protocol (intervention group further categorized by platform usage level: high vs. low).

**Table S13**

*GLMM Summary of AHBS Precautions*

*Fixed Effects Model Test Overview*

| mITT Population (*N* = 55) | | PP Population (*N* = 55) | |
| --- | --- | --- | --- |
| Source | *F* (df1, df2) | Source | *F* (df1, df2) |
| Corrected Model | 1.60 (3, 54) | Corrected Model | 4.01 (5, 16) |
| Timepoint | 0.09 (1, 49) | Timepoint | 0.78 (1, 50) |
| Group × Timepoint | 2.39 (2, 51) | Group × Timepoint | 4.38 (4, 21) |

*Estimated Mean Differences (Post - Pre) by Group*

| Group (*n*) | Difference  (T1 - T0) | Std. Error | *t* (df) | 95% CI | |
| --- | --- | --- | --- | --- | --- |
|  |  |  |  | Lower | Upper |
| Control (29) | -0.59  (10.14 - 10.72) | 0.44 | -1.32 (54) | -1.47 | 0.30 |
| Intervention (26) | 0.77  (10.50 - 9.73) | 0.45 | 1.72 (44) | -0.13 | 1.67 |
| Low Usage (12) | 0.50  (9.42 - 8.92) | 0.85 | 0.59 (104) | -1.19 | 2.19 |
| High Usage (14) | 1.00  (11.43 - 10.43) | 0.38 | 2.65 (6) | 0.09 | 1.91 |

*Estimated Differences Visualized Across Timepoints by Group*

**mITT Population** **PP Population**


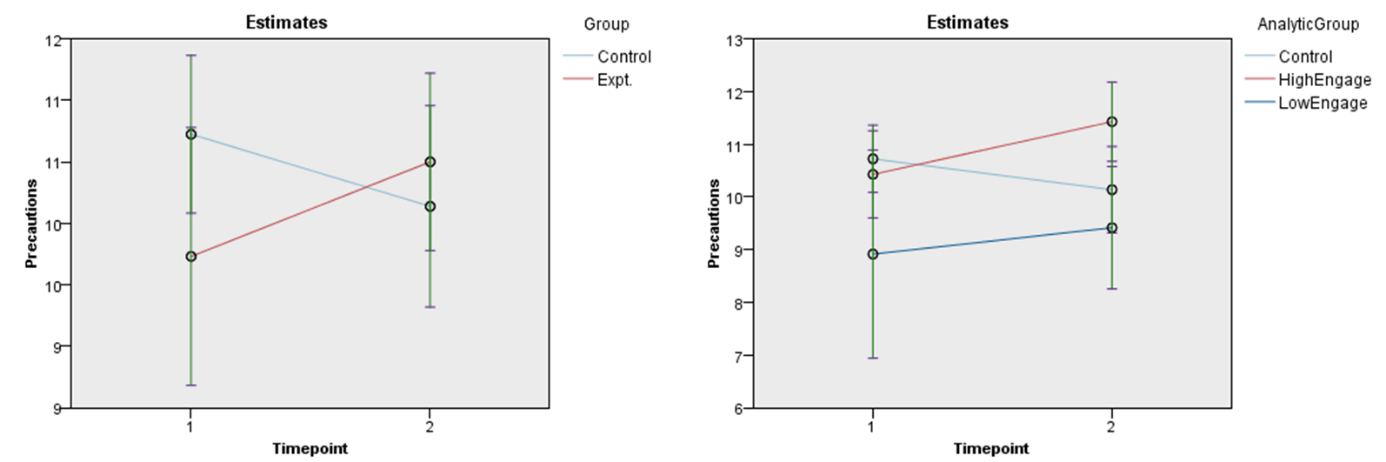


*Note.* GLMM = generalized linear mixed model; mITT = modified intention-to-treat (excluding withdrawals); PP = per protocol (intervention group further categorized by platform usage level: high vs. low).

**Table S14**

*GLMM Summary of Helping Relationships from Significant Others (HRSO)*

*Fixed Effects Model Test Overview*

| mITT Population (*N* = 55) | | PP Population (*N* = 55) | |
| --- | --- | --- | --- |
| Source | *F* (df1, df2) | Source | *F* (df1, df2) |
| Corrected Model | 3.94 (3, 40) | Corrected Model | 2.45 (5, 104) |
| Timepoint | 1.14 (1, 46) | Timepoint | 0.01 (1, 26) |
| Group × Timepoint | 5.90 (2, 49) | Group × Timepoint | 3.01 (4, 19) |

*Estimated Mean Differences (Post - Pre) by Group*

| Group (*n*) | Difference  (T1 - T0) | Std. Error | *t* (df) | 95% CI | |
| --- | --- | --- | --- | --- | --- |
|  |  |  |  | Lower | Upper |
| Control (29) | -8.86  (81.59 - 90.45) | 3.25 | -2.73 (78) | -15.34 | -2.39 |
| Intervention (26) | 4.42  (93.00 - 88.58) | 2.59 | 1.71 (25) | -0.90 | 9.75 |
| Low Usage (12) | 1.50  (90.92 - 89.42) | 2.20 | 0.68 (3) | -5.94 | 8.94 |
| High Usage (14) | 6.93  (94.79 - 87.86) | 4.31 | 1.61 (54) | -1.71 | 15.56 |

*Estimated Differences Visualized Across Timepoints by Group*

**mITT Population** **PP Population**


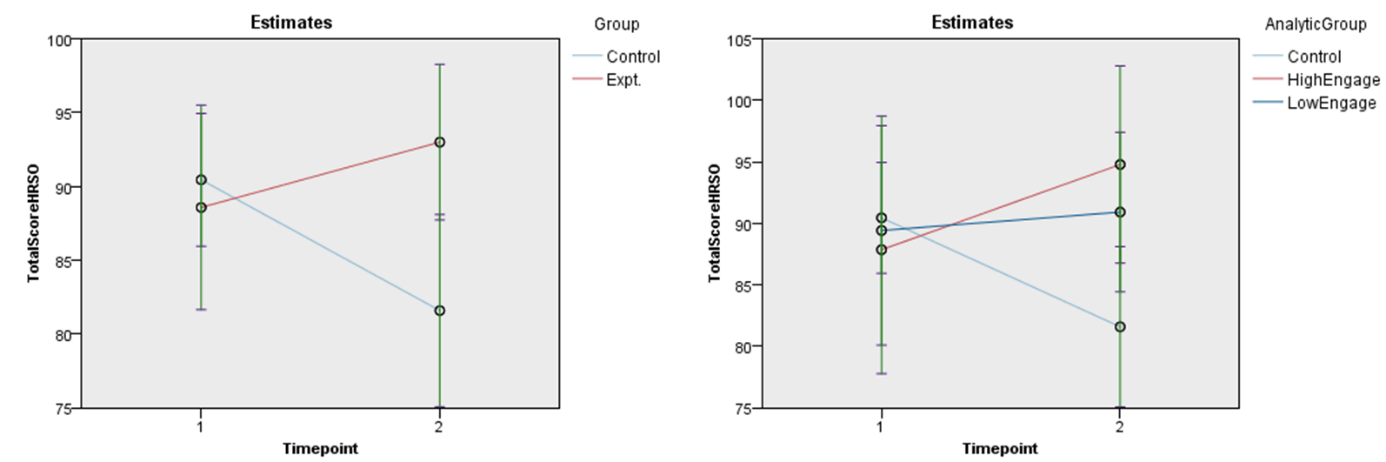


*Note.* GLMM = generalized linear mixed model; mITT = modified intention-to-treat (excluding withdrawals); PP = per protocol (intervention group further categorized by platform usage level: high vs. low).

**Table S15**

*GLMM Summary of HRSO Understanding*

*Fixed Effects Model Test Overview*

| mITT Population (*N* = 55) | | PP Population (*N* = 55) | |
| --- | --- | --- | --- |
| Source | *F* (df1, df2) | Source | *F* (df1, df2) |
| Corrected Model | 3.33 (3, 40) | Corrected Model | 2.34 (5, 14) |
| Timepoint | 2.94 (1, 46) | Timepoint | 0.75 (1, 26) |
| Group × Timepoint | 4.65 (2, 49) | Group × Timepoint | 2.55 (4, 23) |

*Estimated Mean Differences (Post - Pre) by Group*

| Group (*n*) | Difference  (T1 - T0) | Std. Error | *t* (df) | 95% CI | |
| --- | --- | --- | --- | --- | --- |
|  |  |  |  | Lower | Upper |
| Control (29) | -4.17  (31.45 - 35.62) | 1.41 | -2.97 (80) | -6.97 | -1.37 |
| Intervention (26) | 1.12  (35.31 - 34.19) | 1.10 | 1.02 (24) | -1.15 | 3.38 |
| Low Usage (12) | -0.42  (34.00 - 34.42) | 1.16 | -0.36 (6) | -3.23 | 2.39 |
| High Usage (14) | 2.43  (36.43 - 34.00) | 1.70 | 1.43 (39) | -1.01 | 5.86 |

*Estimated Differences Visualized Across Timepoints by Group*

**mITT Population** **PP Population**


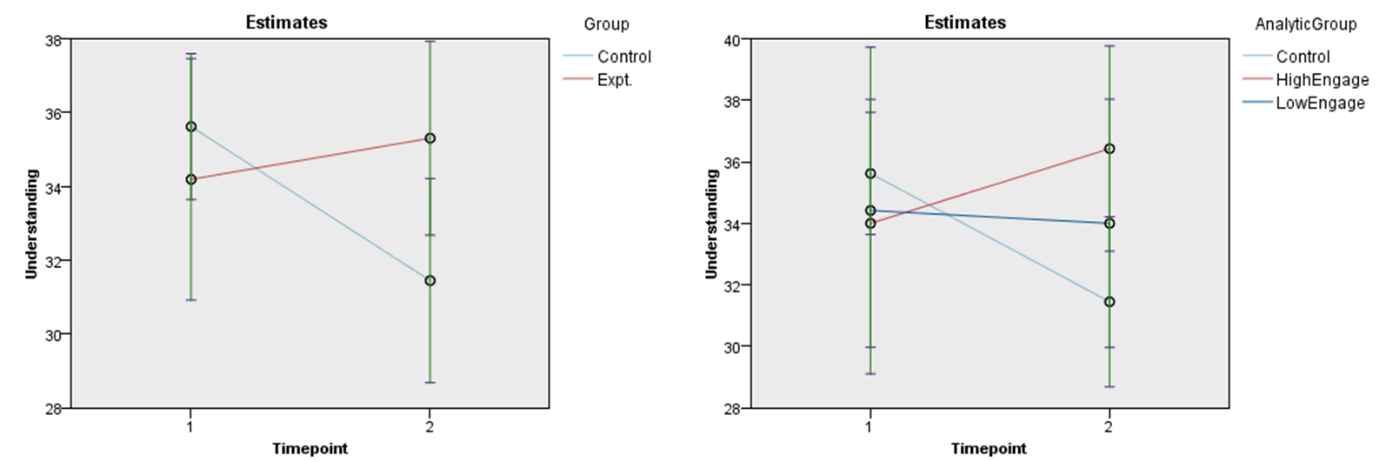


*Note.* GLMM = generalized linear mixed model; mITT = modified intention-to-treat (excluding withdrawals); PP = per protocol (intervention group further categorized by platform usage level: high vs. low).

**Table S16**

*GLMM Summary of HRSO Caring*

*Fixed Effects Model Test Overview*

| mITT Population (*N* = 55) | | PP Population (*N* = 55) | |
| --- | --- | --- | --- |
| Source | *F* (df1, df2) | Source | *F* (df1, df2) |
| Corrected Model | 3.76 (3, 44) | Corrected Model | 2.31 (5, 22) |
| Timepoint | 0.74 (1, 49) | Timepoint | 0.02 (1, 39) |
| Group × Timepoint | 5.58 (2, 49) | Group × Timepoint | 2.88 (4, 30) |

*Estimated Mean Differences (Post - Pre) by Group*

| Group (*n*) | Difference  (T1 - T0) | Std. Error | *t* (df) | 95% CI | |
| --- | --- | --- | --- | --- | --- |
|  |  |  |  | Lower | Upper |
| Control (29) | -3.52  (34.17 - 37.69) | 1.25 | -2.82 (54) | -6.02 | -1.02 |
| Intervention (26) | 2.00  (38.81 - 36.81) | 1.25 | 1.60 (44) | -0.52 | 4.52 |
| Low Usage (12) | 0.92  (38.25 - 37.33) | 1.39 | 0.66 (14) | -2.06 | 3.90 |
| High Usage (14) | 2.93  (39.29 - 36.36) | 1.96 | 1.49 (75) | -0.98 | 6.84 |

*Estimated Differences Visualized Across Timepoints by Group*

**mITT Population** **PP Population**


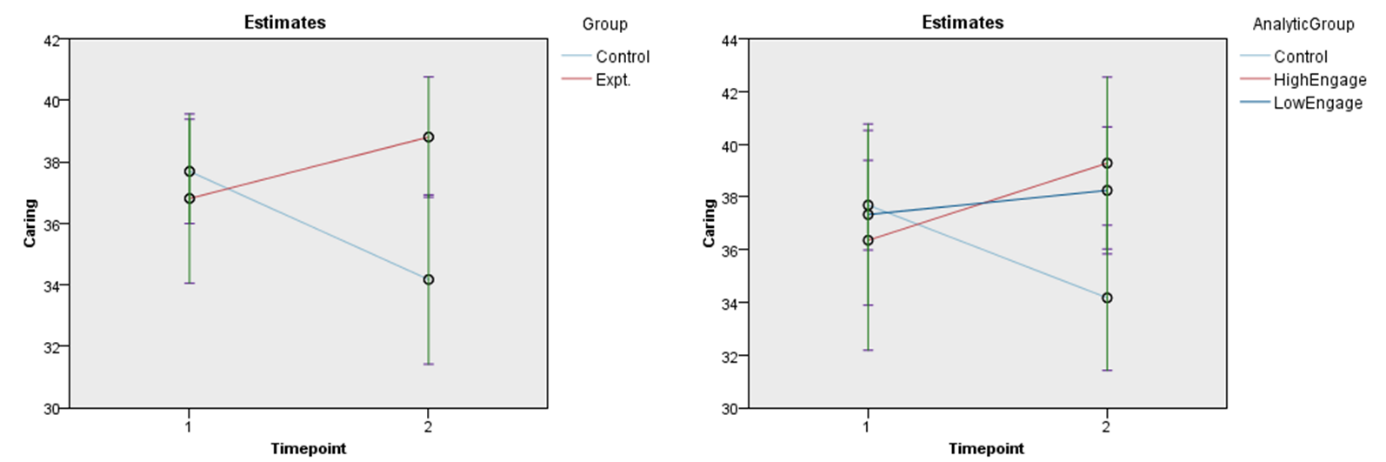


*Note.* GLMM = generalized linear mixed model; mITT = modified intention-to-treat (excluding withdrawals); PP = per protocol (intervention group further categorized by platform usage level: high vs. low).

**Table S17**

*GLMM Summary of HRSO Coaching*

*Fixed Effects Model Test Overview*

| mITT Population (*N* = 55) | | PP Population (*N* = 55) | |
| --- | --- | --- | --- |
| Source | *F* (df1, df2) | Source | *F* (df1, df2) |
| Corrected Model | 4.17 (3, 46) | Corrected Model | 2.65 (5, 37) |
| Timepoint | 0.02 (1, 46) | Timepoint | 0.81 (1, 32) |
| Group × Timepoint | 5.65 (2, 48) | Group × Timepoint | 2.96 (4, 34) |

*Estimated Mean Differences (Post - Pre) by Group*

| Group (*n*) | Difference  (T1 - T0) | Std. Error | *t* (df) | 95% CI | |
| --- | --- | --- | --- | --- | --- |
|  |  |  |  | Lower | Upper |
| Control (29) | -1.17  (15.97 - 17.14) | 0.82 | -1.44 (77) | -2.80 | 0.45 |
| Intervention (26) | 1.31  (18.89 - 17.58) | 0.66 | 1.99 (26) | -0.04 | 2.66 |
| Low Usage (12) | 1.00  (18.67 - 17.67) | 1.02 | 0.98 (30) | -1.08 | 3.08 |
| High Usage (14) | 1.57  (19.07 - 17.50) | 0.84 | 1.86 (19) | -0.19 | 3.34 |

*Estimated Differences Visualized Across Timepoints by Group*

**mITT Population** **PP Population**


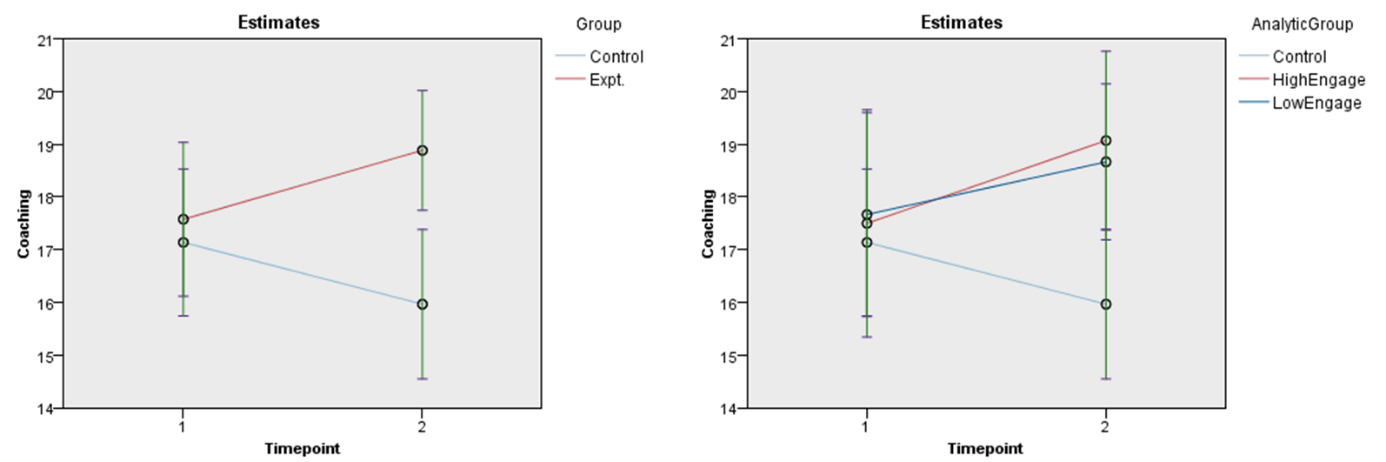


*Note.* GLMM = generalized linear mixed model; mITT = modified intention-to-treat (excluding withdrawals); PP = per protocol (intervention group further categorized by platform usage level: high vs. low).

**Table S18**

*GLMM Summary of Dyadic Adjustment Scale-7 (DAS-7) Total Score*

*Fixed Effects Model Test Overview*

| mITT Population (*N* = 109) | | PP Population (*N* = 109) | |
| --- | --- | --- | --- |
| Source | *F* (df1, df2) | Source | *F* (df1, df2) |
| Corrected Model | 2.31 (4, 43) | Corrected Model | 1.65 (6, 50) |
| Role | 0.45 (1, 51) | Role | 0.44 (1, 51) |
| Timepoint | 0.52 (1, 52) | Timepoint | 1.89 (1, 70) |
| Group × Timepoint | 4.15 (2, 51) | Group × Timepoint | 2.23 (4, 52) |

*Estimated Mean Differences (Post - Pre) by Group*

| Group (*n*) | Difference  (T1 - T0) | Std. Error | *t* (df) | 95% CI | |
| --- | --- | --- | --- | --- | --- |
|  |  |  |  | Lower | Upper |
| Control (58) | -1.47  (20.14 - 21.60) | 0.80 | -1.83 (23) | -3.12 | 0.19 |
| Intervention (51) | 2.49  (23.06 - 20.57) | 1.18 | 2.12 (88) | 0.15 | 4.83 |
| Low Usage (24) | 1.67  (22.21 - 20.54) | 1.77 | 0.94 (93) | -1.85 | 5.18 |
| High Usage (27) | 3.22  (23.80 - 20.59) | 1.55 | 2.07 (74) | 0.12 | 6.31 |

*Estimated Differences Visualized Across Timepoints by Group*

**mITT Population** **PP Population**


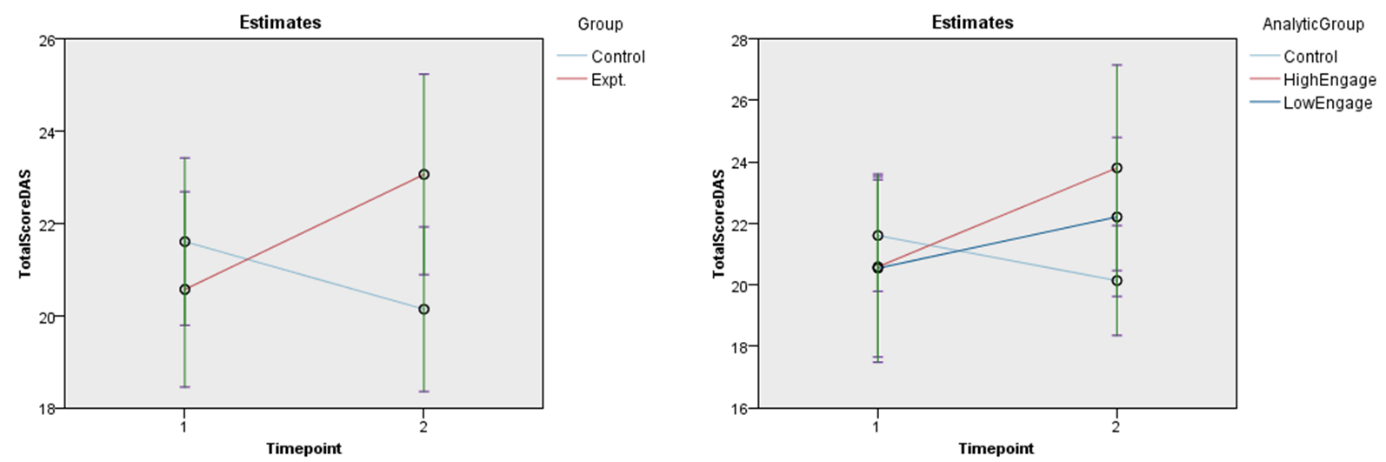


*Note.* GLMM = generalized linear mixed model; mITT = modified intention-to-treat (excluding withdrawals); PP = per protocol (intervention group further categorized by platform usage level: high vs. low).

**Table S19**

*GLMM Summary of WHOQOL-BREF General Quality of Life*

*Fixed Effects Model Test Overview*

| mITT Population (*N* = 109) | | PP Population (*N* = 109) | |
| --- | --- | --- | --- |
| Source | *F* (df1, df2) | Source | *F* (df1, df2) |
| Corrected Model | 0.51 (4, 40) | Corrected Model | 0.97 (6, 32) |
| Role | 0.09 (1, 52) | Role | 0.07 (1, 52) |
| Timepoint | 1.30 (1, 44) | Timepoint | 0.85 (1, 26) |
| Group × Timepoint | 0.44 (2, 48) | Group × Timepoint | 1.32 (4, 42) |

*Estimated Mean Differences (Post - Pre) by Group*

| Group (*n*) | Difference  (T1 - T0) | Std. Error | *t* (df) | 95% CI | |
| --- | --- | --- | --- | --- | --- |
|  |  |  |  | Lower | Upper |
| Control (58) | -0.16  (3.45 - 3.60) | 0.13 | -1.25 (87) | -0.40 | 0.09 |
| Intervention (51) | -0.02  (3.59 - 3.61) | 0.09 | -0.22 (19) | -0.21 | 0.17 |
| Low Usage (24) | -0.08  (3.33 - 3.42) | 0.14 | -0.59 (24) | -0.38 | 0.21 |
| High Usage (27) | 0.04  (3.81 - 3.77) | 0.11 | 0.33 (11) | -0.21 | 0.28 |

*Estimated Differences Visualized Across Timepoints by Group*

**mITT Population** **PP Population**


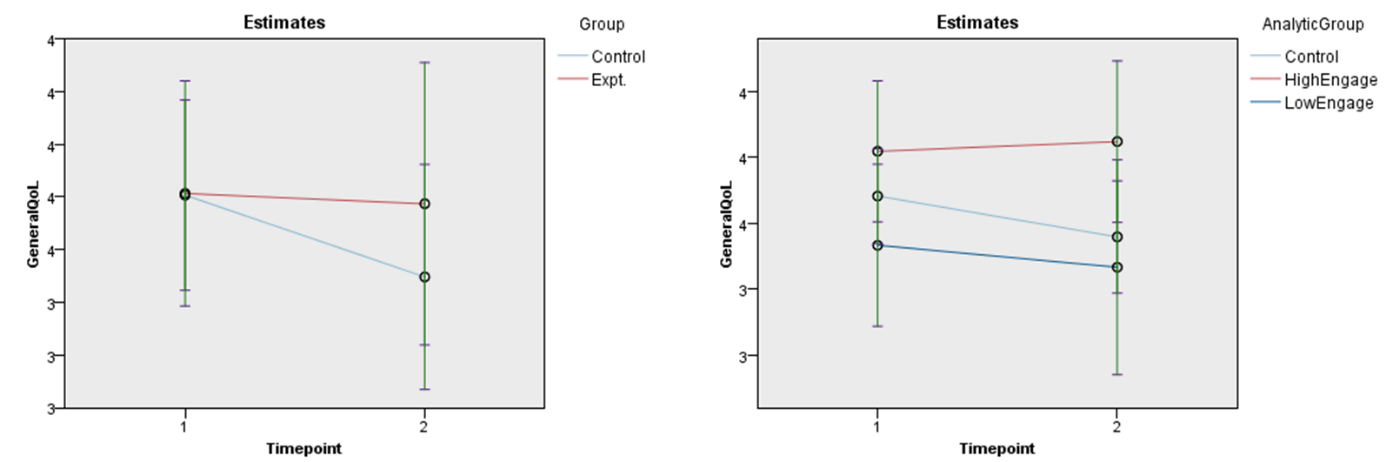


*Note.* GLMM = generalized linear mixed model; mITT = modified intention-to-treat (excluding withdrawals); PP = per protocol (intervention group further categorized by platform usage level: high vs. low).

**Table S20**

*GLMM Summary of WHOQOL-BREF General Health*

*Fixed Effects Model Test Overview*

| mITT Population (*N* = 109) | | PP Population (*N* = 109) | |
| --- | --- | --- | --- |
| Source | *F* (df1, df2) | Source | *F* (df1, df2) |
| Corrected Model | 2.26 (4, 51) | Corrected Model | 2.16 (6, 47) |
| Role | 5.65 (1, 51) | Role | 5.71 (1, 52) |
| Timepoint | 1.93 (1, 49) | Timepoint | 2.84 (1, 46) |
| Group × Timepoint | 0.97 (2, 50) | Group × Timepoint | 1.21 (4, 52) |

*Estimated Mean Differences (Post - Pre) by Group*

| Group (*n*) | Difference  (T1 - T0) | Std. Error | *t* (df) | 95% CI | |
| --- | --- | --- | --- | --- | --- |
|  |  |  |  | Lower | Upper |
| Control (58) | 0.00 (3.02 - 3.02) | 0.12 | 0.00 (52) | -0.24 | 0.24 |
| Intervention (51) | 0.23 (3.16 - 2.93) | 0.12 | 1.94 (47) | -0.01 | 0.48 |
| Low Usage (24) | 0.08 (3.00 - 2.92) | 0.19 | 0.43 (67) | -0.30 | 0.47 |
| High Usage (27) | 0.37 (3.31 - 2.94) | 0.14 | 2.62 (24) | 0.08 | 0.65 |

*Estimated Differences Visualized Across Timepoints by Group*

**mITT Population** **PP Population**


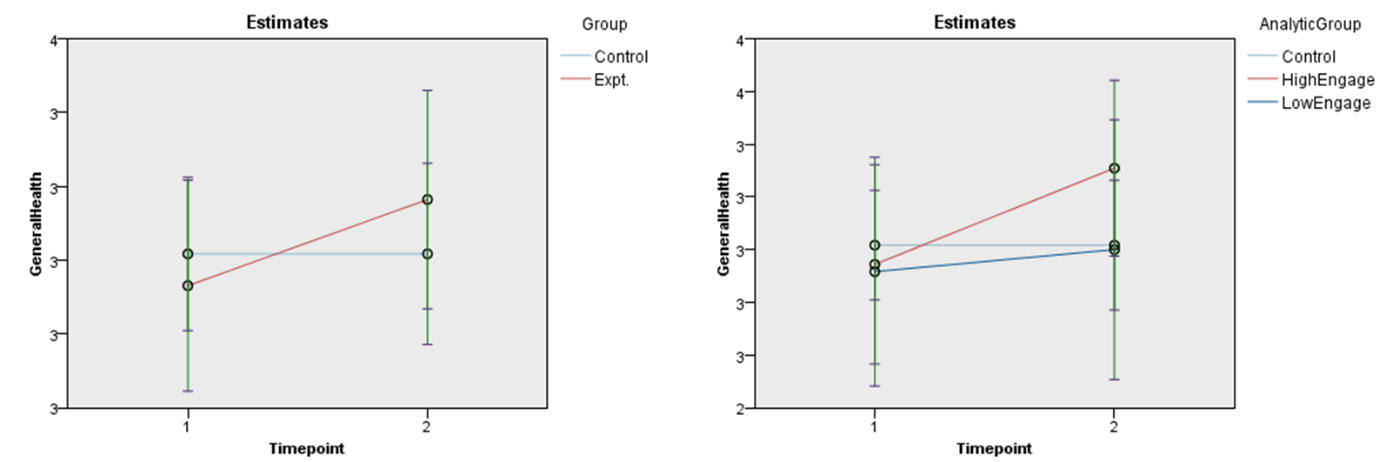


*Note.* GLMM = generalized linear mixed model; mITT = modified intention-to-treat (excluding withdrawals); PP = per protocol (intervention group further categorized by platform usage level: high vs. low).

**Table S21**

*GLMM Summary of WHOQOL-BREF Physical*

*Fixed Effects Model Test Overview*

| mITT Population (*N* = 109) | | PP Population (*N* = 109) | |
| --- | --- | --- | --- |
| Source | *F* (df1, df2) | Source | *F* (df1, df2) |
| Corrected Model | 3.73 (4, 58) | Corrected Model | 3.33 (6, 47) |
| Role | 10.64 (1, 51) | Role | 10.82 (1, 52) |
| Timepoint | 0.33 (1, 48) | Timepoint | 0.04 (1, 38) |
| Group × Timepoint | 2.30 (2, 50) | Group × Timepoint | 3.23 (4, 45) |

*Estimated Mean Differences (Post - Pre) by Group*

| Group (*n*) | Difference  (T1 - T0) | Std. Error | *t* (df) | 95% CI | |
| --- | --- | --- | --- | --- | --- |
|  |  |  |  | Lower | Upper |
| Control (58) | -0.54  (13.94 - 14.48) | 0.29 | -1.84 (62) | -1.13 | 0.05 |
| Intervention (51) | 0.31  (14.39 - 14.08) | 0.27 | 1.15 (36) | -0.24 | 0.87 |
| Low Usage (24) | 0.62  (13.71 - 13.10) | 0.42 | 1.47 (44) | -0.23 | 1.47 |
| High Usage (27) | 0.04  (14.99 - 14.95) | 0.34 | 0.12 (24) | -0.66 | 0.74 |

*Estimated Differences Visualized Across Timepoints by Group*

**mITT Population** **PP Population**


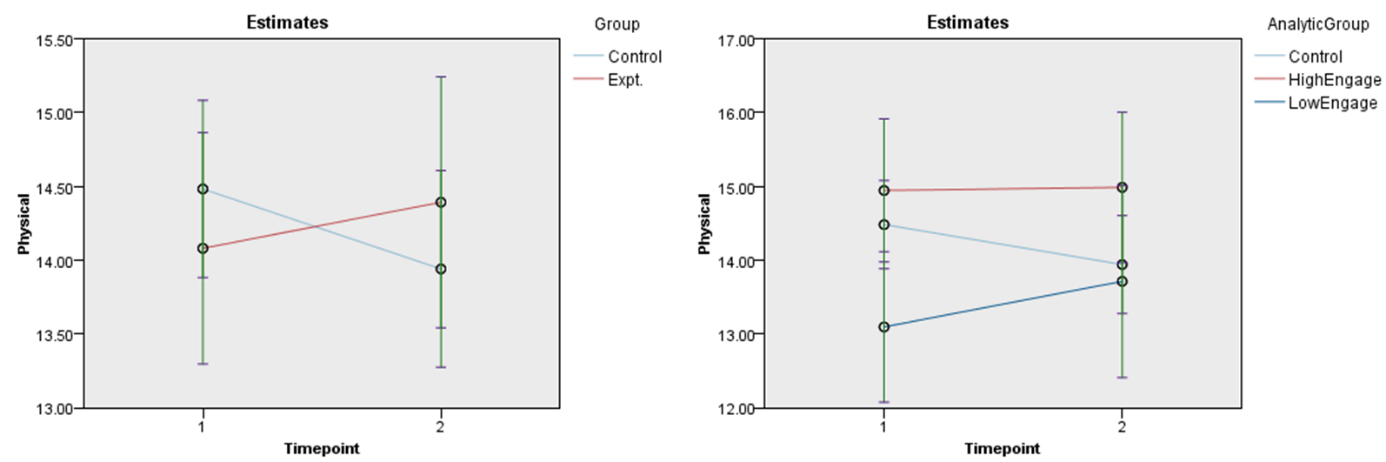


*Note.* GLMM = generalized linear mixed model; mITT = modified intention-to-treat (excluding withdrawals); PP = per protocol (intervention group further categorized by platform usage level: high vs. low).

**Table S22**

*GLMM Summary of WHOQOL-BREF Psychological*

*Fixed Effects Model Test Overview*

| mITT Population (*N* = 109) | | PP Population (*N* = 109) | |
| --- | --- | --- | --- |
| Source | *F* (df1, df2) | Source | *F* (df1, df2) |
| Corrected Model | 1.13 (4, 43) | Corrected Model | 1.54 (6, 28) |
| Role | 0.32 (1, 52) | Role | 0.34 (1, 52) |
| Timepoint | 0.91 (1, 44) | Timepoint | 0.12 (1, 23) |
| Group × Timepoint | 2.01 (2, 47) | Group × Timepoint | 2.11 (4, 29) |

*Estimated Mean Differences (Post - Pre) by Group*

| Group (*n*) | Difference  (T1 - T0) | Std. Error | *t* (df) | 95% CI | |
| --- | --- | --- | --- | --- | --- |
|  |  |  |  | Lower | Upper |
| Control (58) | -0.53  (13.14 - 13.67) | 0.31 | -1.70 (95) | -1.15 | 0.09 |
| Intervention (51) | 0.17  (14.00 - 13.83) | 0.21 | 0.81 (15) | -0.28 | 0.62 |
| Low Usage (24) | 0.28  (13.22 - 12.94) | 0.30 | 0.92 (14) | -0.37 | 0.93 |
| High Usage (27) | 0.07  (14.67 - 14.60) | 0.29 | 0.26 (15) | -0.54 | 0.69 |

*Estimated Differences Visualized Across Timepoints by Group*

**mITT Population** **PP Population**


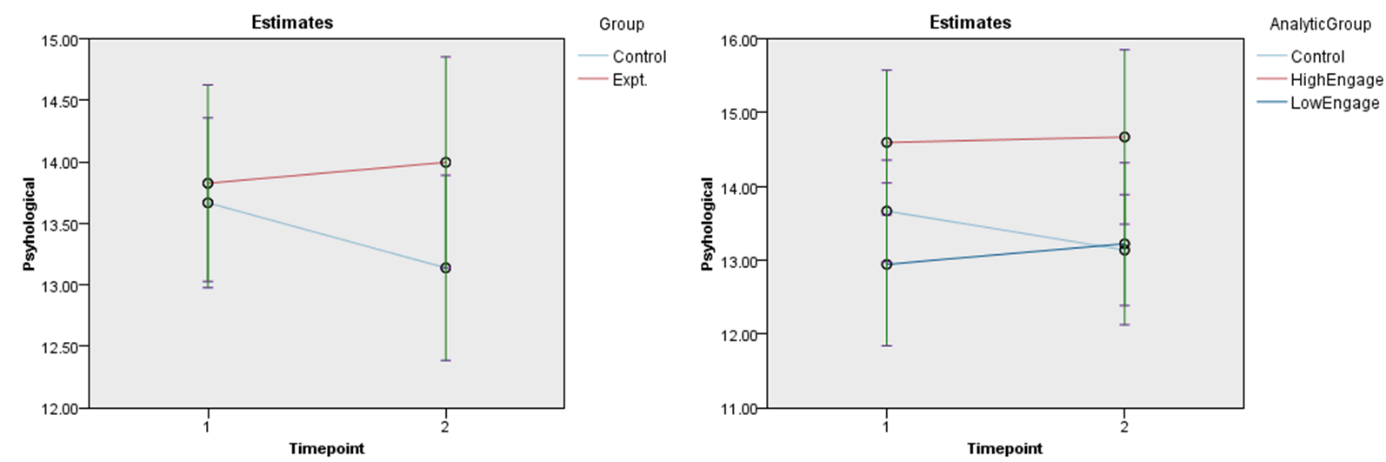


*Note.* GLMM = generalized linear mixed model; mITT = modified intention-to-treat (excluding withdrawals); PP = per protocol (intervention group further categorized by platform usage level: high vs. low).

**Table S23**

*GLMM Summary of WHOQOL-BREF Social*

*Fixed Effects Model Test Overview*

| mITT Population (*N* = 109) | | PP Population (*N* = 109) | |
| --- | --- | --- | --- |
| Source | *F* (df1, df2) | Source | *F* (df1, df2) |
| Corrected Model | 0.55 (4, 55) | Corrected Model | 0.57 (6, 44) |
| Role | 1.25 (1, 51) | Role | 1.26 (1, 51) |
| Timepoint | 0.02 (1, 47) | Timepoint | 0.08 (1, 37) |
| Group × Timepoint | 0.67 (2, 48) | Group × Timepoint | 0.50 (4, 42) |

*Estimated Mean Differences (Post - Pre) by Group*

| Group (*n*) | Difference  (T1 - T0) | Std. Error | *t* (df) | 95% CI | |
| --- | --- | --- | --- | --- | --- |
|  |  |  |  | Lower | Upper |
| Control (58) | -0.26  (13.91 - 14.17) | 0.30 | -0.88 (63) | -0.85 | 0.33 |
| Intervention (51) | 0.20  (14.14 - 13.94) | 0.27 | 0.75 (35) | -0.34 | 0.75 |
| Low Usage (24) | 0.42  (14.04 - 13.63) | 0.41 | 1.01 (40) | -0.42 | 1.25 |
| High Usage (27) | 0.02  (14.23 - 14.22) | 0.35 | 0.04 (26) | -0.69 | 0.72 |

*Estimated Differences Visualized Across Timepoints by Group*

**mITT Population** **PP Population**


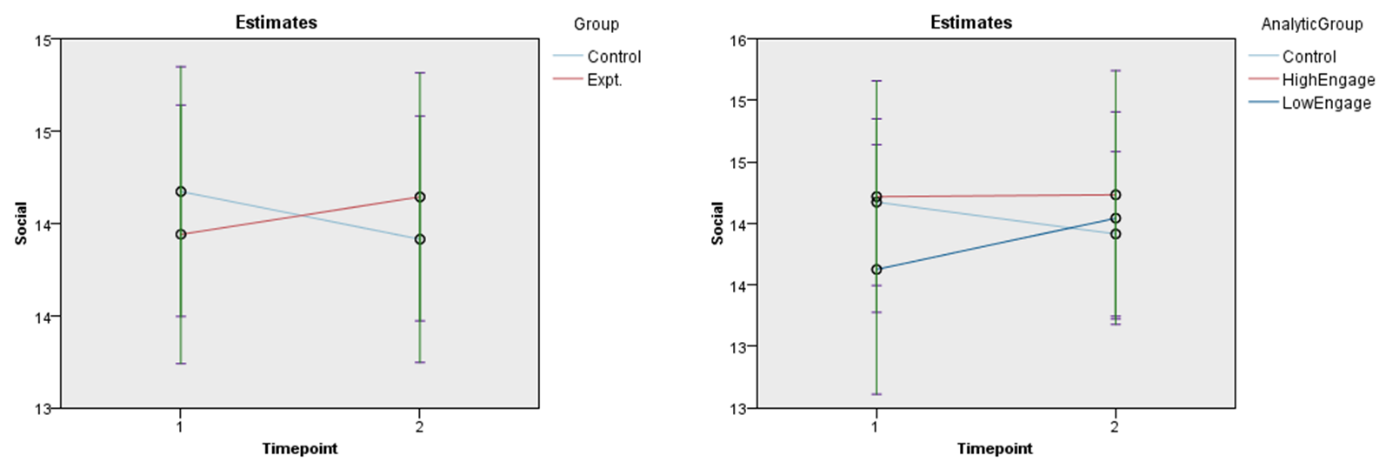


*Note.* GLMM = generalized linear mixed model; mITT = modified intention-to-treat (excluding withdrawals); PP = per protocol (intervention group further categorized by platform usage level: high vs. low).

**Table S24**

*GLMM Summary of WHOQOL-BREF Environment*

*Fixed Effects Model Test Overview*

| mITT Population (*N* = 109) | | PP Population (*N* = 109) | |
| --- | --- | --- | --- |
| Source | *F* (df1, df2) | Source | *F* (df1, df2) |
| Corrected Model | 1.13 (4, 51) | Corrected Model | 0.96 (6, 57) |
| Role | 0.76 (1, 52) | Role | 0.78 (1, 51) |
| Timepoint | 1.15 (1, 50) | Timepoint | 0.26 (1, 46) |
| Group × Timepoint | 1.16 (2, 49) | Group × Timepoint | 0.83 (4, 53) |

*Estimated Mean Differences (Post - Pre) by Group*

| Group (*n*) | Difference  (T1 - T0) | Std. Error | *t* (df) | 95% CI | |
| --- | --- | --- | --- | --- | --- |
|  |  |  |  | Lower | Upper |
| Control (58) | -0.54  (14.28 - 14.82) | 0.28 | -1.91 (47) | -1.10 | 0.03 |
| Intervention (51) | 0.09  (14.68 - 14.58) | 0.31 | 0.30 (52) | -0.52 | 0.70 |
| Low Usage (24) | 0.24  (14.41 - 14.17) | 0.39 | 0.61 (29) | -0.56 | 1.04 |
| High Usage (27) | -0.04  (14.91 - 14.95) | 0.46 | -0.09 (71) | -0.95 | 0.87 |

*Estimated Differences Visualized Across Timepoints by Group*

**mITT Population** **PP Population**


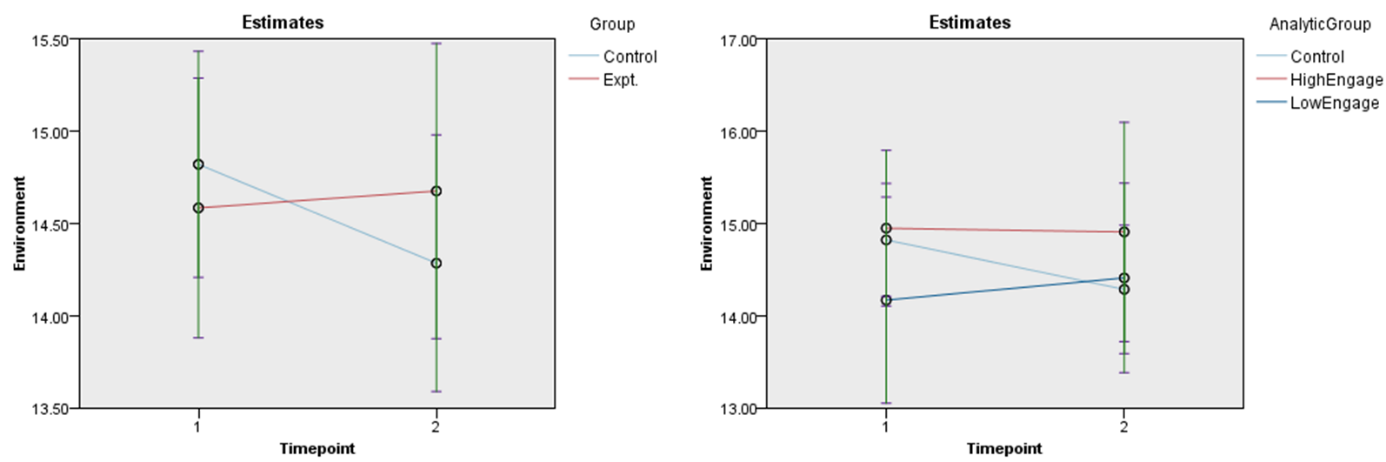


*Note.* GLMM = generalized linear mixed model; mITT = modified intention-to-treat (excluding withdrawals); PP = per protocol (intervention group further categorized by platform usage level: high vs. low).
